# *BCL11A* and *HBS1L-MYB* polymorphisms, in association with hydroxyurea and sex, modulate fetal hemoglobin levels in individuals with sickle cell anemia in the western region of Bahia, Brazil

**DOI:** 10.1007/s11033-026-12151-9

**Published:** 2026-06-23

**Authors:** Manoel Ferreira de Magalhães Filho, Ilana Luize Rocha Santana, Pâmela Lourdes Pereira da Silva, Larissa Paola Rodrigues Venancio

**Affiliations:** 1 Centro das Ciências Biológicas e da Saúde (CCBS), Federal University of Western Bahia (UFOB), Barreiras, 47808-021 Bahia Brazil; 2 Instituto de Biociências, Letras e Ciências Exatas (IBILCE), São Paulo State University (UNESP), São José do Rio Preto, São Paulo, 15054-000 Brazil

**Keywords:** Sickle cell anemia, Fetal hemoglobin modulation, Hydroxyurea response, *BCL11A*, *HBS1L-MYB*, Genetic modifiers

## Abstract

**Background:**

Sickle cell anemia (SCA) is a condition caused by a mutation in the *HBB* gene, leading to the production of hemoglobin S in red blood cells. Hydroxyurea (HU), used in treatment, increases fetal hemoglobin (HbF) levels, thereby reducing erythrocyte sickling, as do single-nucleotide polymorphisms (SNPs) in the *BCL11A* gene and the *HBS1L-MYB* intergenic region. In this study, we investigated the combined effects of HU therapy, β^S^-globin haplotypes, and SNPs in *BCL11A* and the *HBS1L-MYB* intergenic region on HbF levels in a cohort from Western Bahia, Brazil.

**Methods and results:**

The DNA of 43 individuals with SCA was analyzed for SNPs rs4671393, rs7557939, rs1427407, and rs11886868 (*BCL11A*), and rs4895441, rs9402686, and rs11759553 (*HBS1L-MYB*). Individuals receiving HU therapy presented significantly higher HbF levels than untreated individuals (*p* = 0.028). Factorial ANOVA demonstrated a significant interaction between HU use, β^S^ haplotypes, and sex on HbF variability (*p* < 0.001). Significant associations with HbF levels were observed for rs7557939 in *BCL11A* under overdominant models in untreated individuals, and for rs11759553, rs4895441, and rs9402686 in the *HBS1L-MYB* region under dominant and recessive models. Multiple linear regression indicated that HU use, sex, rs1427407, rs7557939, and rs4895441 explained 46.5% of the variability in HbF (R² = 0.465; *p* = 0.001). Linkage disequilibrium (LD) analyses revealed non-random associations between rs4671393 and rs1427407; and between rs9402686, rs4895441, and rs11759553. The transcription factors GATA-1, SMARCA4, and CBFA2T3, which are essential for regulating hematopoietic cell production and differentiation, were associated with these variants.

**Conclusions:**

These findings reinforce the contribution of genetic variants in *BCL11A* and *HBS1L-MYB* to HbF modulation in SCA. The identification of genetic markers associated with increased HbF levels may contribute to future strategies for individualized therapeutic monitoring in SCA.

**Supplementary Information:**

The online version contains supplementary material available at 10.1007/s11033-026-12151-9.

## Introduction

Sickle cell anemia (SCA) is a genetic hematological disorder that affects thousands of people worldwide [1]. It is characterized by the homozygous presence of the β^S^ allele caused by a point mutation in the β-globin gene (HBB: c.20 A > T; rs334), resulting in the production of hemoglobin S (HbS), which, under deoxygenation conditions, undergoes polymerization, leading to a reduction in the deformability of erythrocytes and the characteristic sickling of red blood cells [2].

The condition leads to signs and symptoms that may include chronic anemia, jaundice, pain crises, and aseptic necrosis due to vaso-occlusive events, fatigue, splenic sequestration, acute chest syndrome, immunodeficiency, increased risk of stroke, as well as permanent vision damage [3].

The drug conventionally standardized by the United States Food and Drug Administration (US FDA) for the treatment of the disease is hydroxyurea (HU), which aims to increase fetal hemoglobin (HbF), thereby reducing HbS polymerization and erythrocyte density and inhibiting the sickling of red blood cells [4]. However, the effects of HU remain heterogeneous, and there is no concrete explanation for this event [5]. Despite these gaps, research points to a strong relationship between the presence of polymorphisms in genes related to HbF modulation and the response to HU treatment, including the B-cell lymphoma/leukemia 11 A gene (*BCL11A*) and the region between the HBS1-like translational GTPase and v-myb avian myeloblastosis viral homolog genes (*HBS1L-MYB*) [6–9].

*BCL11A* (2p16) is a gene that encodes the BCL11A transcription factor, involved in hemoglobin switching, which acts as a negative modulator of fetal hemoglobin levels in the erythroid cells of adult individuals [10]. Increased HbF levels have been widely associated with a milder clinical presentation of sickle cell disease due to their inhibitory effect on erythrocyte sickling [11]. Studies have linked single-nucleotide polymorphisms (SNPs) in the BCL11A gene to modulation of HbF expression [12].

Similarly, a region on the long arm of chromosome 6, between the HBS1L and MYB genes, is also involved in the increase in HbF. The *HBS1L* gene was first described in a study aimed at identifying pancreatic oncogenes, and its role in erythrocyte proliferation remains poorly understood [13]. The *MYB* gene is described as a proto-oncogene, a regulator of the production and differentiation of stem and progenitor cells in human bone marrow, and is mainly involved in hematopoietic disorders [14]. Data indicates that SNPs residing in the region between these two genes may be important modulators of HbF expression, further contributing to the development of SCA treatment [15].

In this study, we evaluated the association between polymorphisms in *BCL11A* (rs4671393, rs1427407, rs7557939, and rs11886868) and in the *HBS1L-MYB* intergenic region (rs9402686, rs4895441, and rs11759553) on HbF variation, considering haplotypes and HU use. The SNPs were selected based on previously reported evidence demonstrating their functional relevance in HbF regulation and their association with clinical variability and response to hydroxyurea treatment [16–20].

## Materials and methods

### Biological sample

For this study, 10 mL of peripheral blood was collected from 43 individuals with SCA treated at Barreiras’ Sickle Cell Disease Program (Western Bahia), based at the Leonídia Ayres de Almeida Municipal Basic Health Unit, Western Bahia, Brazil, and divided according to HU use (HU+) or non-use (HU-). The samples were transported under refrigeration to the Infectious Agents and Vectors Laboratory (LAIVE) at the Federal University of Western Bahia, where they were used for leukocyte genomic DNA extraction with Chelex^®^ 5% reagent and for confirmation of homozygous HbS by PCR-RFLP [21]. HbF levels were obtained from the patients’ medical records. The values correspond to laboratory results determined by HPLC and recorded during routine clinical evaluations in the medical records.

Given that HbF levels can be influenced by the β^S^ haplotype profile, the patients included in this study were also evaluated for their haplotype profile and its relationship with hematological and biochemical parameters, as well as with the use or non-use of HU; these data have been previously described [22]. β^S^ haplotype profiles were determined by PCR-RFLP [23], and atypical haplotypes were characterized as described in the literature [24].

The hematological data were obtained from laboratory records available in the patients’ medical charts, including routine tests performed during clinical follow-up. The sample excluded individuals who had received a blood transfusion in the 3 months prior to the day of collection, individuals in pain crisis, those who had undergone bone marrow transplantation, pregnant women, those using medications that alter hematological parameters, related individuals, and those who did not have data in their medical records regarding HbF levels. All experimental procedures were approved and registered by the research ethics committee of the Federal University of Western Bahia (UFOB) under number CAAE 29075620.6.0000.5026 in accordance with the National Health Council resolution number 466/2012 (Ministry of Health-Brazil).

### Genotyping of the *BCL11A* gene SNPs

SNPs rs4671393 (A > G), rs7557939 (G > A), and rs1427407 (T > G) were genotyped using the TaqMan^®^ system (Applied Biosystems), using the TaqPath™ ProAmp™ Master Mix PCR kit and analyzed in a QuantStudio 5 thermocycler (Applied Biosystems). The SNP rs11886868 (T > C) was genotyped by Tetra-Primer ARMS-PCR, designed for this study [25], under the following reaction conditions: 1X buffer, MgCl_2_ at 6mM, dNTPs at 0.5 mM, Forward outer, Reverse outer, Forward inner, and Reverse inner primers (Supplementary Table S1) at 0.2 µM each, DNA at 100 ng/µL, and ultrapure water to a final volume of 10 µL. The reactions were performed in a SimpliAmp thermal cycler (Applied Biosystems). The amplification product was analyzed by 2% agarose gel electrophoresis and stained with ethidium bromide.

### Genotyping of SNPs in the *HBS1L-MYB* intergenic region

PCR-RFLP was used to detect rs4895441 (A > G). The reactions were performed to a final volume of 25 µL using 100 ng of genomic DNA, 1X PCR Buffer, 0.5 mM of each dNTP (Invitrogen), 0.1 U/µL of Platinum™ Taq DNA Polymerase (Invitrogen), and oligonucleotide primers at a concentration of 0.2 µM [26]. The amplification products were analyzed on a 2% agarose gel and stained with ethidium bromide. The RsaI enzyme was used to digest rs4895441 according to the manufacturer’s specifications, and the digestion products were visualized on a 3% agarose gel stained with ethidium bromide. The SNPs rs9402686 (G > A) and rs11759553 (A > T) were detected by the TaqMan^®^ system (Applied Biosystems), using the TaqPath™ ProAmp™ Master Mix PCR reaction kit and analyzed in a QuantStudio 5 thermocycler (Applied Biosystems).

### Statistical analysis

Quantitative data were initially evaluated for normality and homogeneity of variances using the Shapiro-Wilk and Levene tests, respectively. Categorical variables, including sex and polymorphism frequencies, were evaluated using Pearson’s chi-square or Fisher’s exact tests. Hardy-Weinberg equilibrium (HWE) was assessed using HW_TEST software (version 1.1). HbF levels were also compared using genetic models: dominant, recessive, allelic, additive and overdominant [27–29], in this case, comparisons between two groups were performed using the t-student test or Mann-Whitney U test, while comparisons between three or more groups were conducted using the Kruskal-Wallis’s test or one-way ANOVA. Linear regression analysis was used to assess the influence of sex, use or non-use of HU, and the other variables on HbF levels. Correlation analyses were performed using Pearson’s correlation coefficients. A two-way ANOVA was performed to assess the interaction effects between variables on HbF levels. A square root transformation (sqrt transformation) was applied to the HbF values prior to the parametric analyses. Data that followed a normal distribution were described as mean ± SD, and those that did not follow a normal distribution were described as median and minimum and maximum values. For all analyses, the significance level used was *p* < 0.05 and was analyzed using Jamovi software (version 2.3).

Since linkage disequilibrium analysis depends on the physical proximity of variants to estimate non-random allelic associations and define haplotype blocks [30], the physical genomic positions of the SNPs were initially obtained using the online tool e! GRCh37 (https://grch37.ensembl.org/index.html). Subsequently, LD analysis was performed using Haploview version 4.2 software to calculate the LD coefficient (D’) and to construct haplotype block maps of the investigated variants. Finally, data related to the genomic location and functionality of the variants were found in the HaploReg version 4.2 (https://pubs.broadinstitute.org/mammals/haploreg/haploreg_v4.1.php) and RegulomeDB version 2.2 (https://regulomedb.org/regulome-search) databases.

## Results

### Characterization of the group, β^S^ haplotype, HWE, genotype, and allele frequency

The general characterization of the groups investigated in the study is in Table 1. The median age is 27 years for individuals who use HU (HU+) and 20 years for those who do not (HU-). HbF levels do not correlate with the age of the study participants (Pearson’s *r* = 0.160, *p* = 0.305). The HU+ group had a mean HbF% of 17.3% ± 7.89, and the HU- group had a mean HbF% of 11.7% ± 7.95. These data indicate that SS individuals using HU have significantly higher HbF values than individuals who do not use the medication (t-test = 2.28; *p* = 0.028).

The individuals were also evaluated regarding the β^S^ haplotype profile, and the data are also described in Table 1. All participants in the study have Bantu and Benin chromosomes in a homozygous or heterozygous state, and only two carry chromosomes with an atypical haplotype profile (according to the classification described by Okumura et al., 2019 [24]). Therefore, none of the participants exhibits the XmnI/HBG2 (11p15.4) polymorphism, an important modifier that influences increased HbF levels [31–32].

A factorial ANOVA analysis indicated an interaction between HU use, haplotype profile, and sex on the HbF levels of the individuals studied (general model: F = 4.462; df = 11; *p* < 0.001). The model indicated an interaction between haplotype profile and HU use on HbF levels (F = 8.1482; df = 2; *p* = 0.002), with Benin/Benin HU- individuals presenting higher HbF levels compared to Bantu/Bantu HU- individuals (F = 15.35, df = 1; *p* = 0.0078), Bantu/Bantu HU+ individuals having higher HbF levels than Bantu/Bantu HU- (F = 22.9075; df = 1; *p* = 0.00029), and Bantu/Bantu HU- individuals with lower HbF levels than Bantu/Benin HU- (F = 8.9389, df = 1; *p* = 0.015). Therefore, overall, the data indicated that Bantu/Bantu individuals who do not use HU have the lowest HbF levels among the groups.

When gender was included in the analysis, the model remained significant (F = 7.638; df = 2; *p* = 0.0002). In general, females have higher levels of HbF than males. It should be noted that female individuals who use HU and have the Bantu/Bantu haplotype profile have approximately 3.8-fold higher HbF levels than men who do not use HU and who are homozygous for the Bantu haplotype (*p* = 0.006).

**Table 1:** Descriptive analysis of the individuals evaluated

	HU+ patients (*N* = 26)	HU- patients (*N* = 17)	*p*-value
	Mean ± SD	Mean ± SD	
Age (years)	27.9 ± 12.78	21.8 ± 13.87	
Min – Max	8–49	4–56	0.145
*Gender*			
Male, n (%)	11 (42.30%)	9 (52.95%)	
Female, n (%)	15 (57.70%)	8 (47.05%)	0.545
*Hemoglobin*			
HbF (%)	17.3 ± 7.89	11.7 ± 7.95	**0.028** ^*^
Med	17.3	10.8	
Min – Max	3.7–32	2–29.5	
*HbF by gender*			
Male (%)	17.6 ± 9.07	7.34 ± 4.56	0.005*
Med	19	6.6	
Min – Max	3.7–29.3	2–17.3	
*Female (%)*			
Med	17.1 ± 7.24	16.6 ± 8.30	0.879
Min – Max	16.3	14.5	
	6–32	3.5–29.5	
White blood cells (mm³)	7.539 ± 1.71	11.521 ± 1.16	0.011*
Eosinophils (mm³)	315 ± 250	667 ± 719	0.073
Segmented cells (mm³)	4.001 ± 2542	6.078 ± 4202	0.067
Lymphocytes (mm³)	3.210 ± 1.513	3.987 ± 2.139	0.195
Monocytes (mm³)	722 ± 680	1.176 ± 661	0.055
Platelets (mm³)	338.132.000 ± 183.287	382.253.000 ± 164.786	0.401
*Haplotypes*			
Bantu/Bantu	11 (42.3%)	5 (29.4%)	
Bantu/Benin	10 (38.46%)	6 (35,3%)	
Benin/Benin	4 (15.3%)	3 (17,6%)	
Bantu/Atypical 1	0 (0%)	1 (5.9%)	
Bantu/Atypical 2	0 (0%)	1 (5.9%)	

SD = standard deviation, HbF = fetal hemoglobin, HU + = uses hydroxyurea, HU- = does not use hydroxyurea, Min = minimum, Max = maximum, Med = median, p* = <0.05 (values in bold indicate significant difference)

Therapeutic adherence among patients taking hydroxyurea (HU+) was inferred from clinical follow-up records and the observation of leukopenia, which is commonly seen in those taking the medication [33, 34]. HbF levels were initially compared between the HU + and HU- groups, without accounting for interactions among genetic variants, to evaluate drug response independently; higher HbF levels were observed in the HU+ group (Table 1, *p* = 0.028). Analysis by sex demonstrated a significant difference in HbF levels between males (*p* = 0.005), with individuals in the HU+ group having a higher mean HbF level than in the HU− group, corresponding to an increase of approximately 240%. The allele and genotype frequencies for each SNP were evaluated in the HU + and HU- groups using Pearson’s chi-square test, which revealed no significant differences between the groups (Table 2). HWE data showed that all SNPs were in equilibrium.

**Table 2:** Genotype and allele frequencies, frequency analyses, HWE, and Pearson’s χ2 for the SNPs investigated

Gene		SNPs	Genotype / Allele	Frequency	HWE (*p*)	HWE χ2	Pearson χ2
*BCL11A*	Genotype frequency	rs4671393	GG	0.512			
			AA	0.046	0.402	0.7	0.398
			AG	0.442			
		rs1427407	GG	0.488			
			TT	0.024	0.106	2.61	0.681
			TG	0.488			
		rs7557939	GG	0.186			
			AA	0.232	0.278	1.18	0.32
			GA	0.582			
		rs11886868	TT	0.698			
			CC	0.069	0.128	2.32	0.737
			TC	0.233			
	Allele frequency	rs4671393 Minor allele: A	G	0.732			
			A	0.268			
		rs1427407 Minor allele: T	G	0.732			
			T	0.268			
		rs7557939 Minor allele: G	G	0.477			
			A	0.523			
		rs11886868 Minor allele: C	T	0.814			
			C	0.186			
*HBS1L-MYB*	Genotype frequency	rs9402686	GG	0.673	0.204	1.61	0.165
			AA	0.058			
			AG	0.269			
		rs4895441	GG	0.058	0.073	3.21	0.173
			AA	0.692			
			AG	0.25			
		rs11759553	AA	0.488	0.589	0.29	0.544
			AT	0.396			
			TT	0.116			
	Allele frequency	rs9402686 Minor allele: A	G	0.8			
			A	0.2			
		rs4895441 Minor allele: G	G	0.18			
			A	0.82			
		rs11759553 Minor allele: T	A	0.68			
			T	0.32			

HWE = Hardy-Weinberg equilibrium, SNP = single nucleotide polymorphism

### Comparative analysis of HbF values and *BCL11A* SNPs

The analysis of the percentage variation of HbF according to the genetic models for all SNPs evaluated was performed considering the following criteria: (1) All individuals analyzed, regardless of sex and use of HU; (2) Use or non-use of HU; (3) Based on sex, regardless of the use of HU. The data for the analysis of *BCL11A* gene SNPs for each criterion are presented in Supplementary Table S2.

#### Criterion 1

As shown in Table S2, no statistically significant differences in HbF levels were observed for any SNP under the dominant model in Criterion 1.

#### Criterion 2

**Fig. 1:**
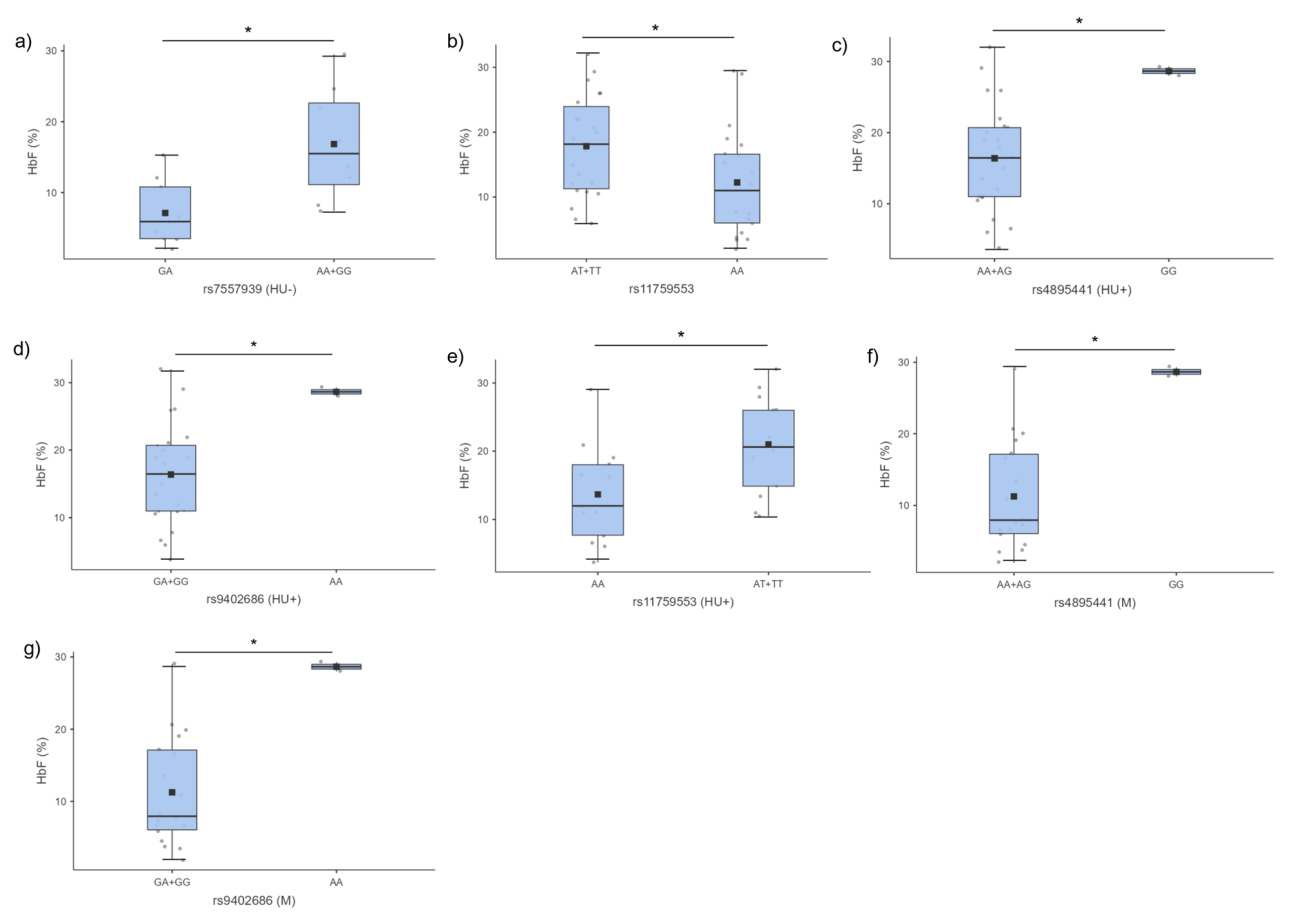
Analysis of the different dominance models applied to SNPs of the *BCL11A* gene and the *HBS1L-MY*B intergenic region **a**) Comparison between HbF levels and the overdominant model for SNP rs7557939 in the HU- group, **b**) HbF levels and the recessive model for SNP rs11759553, **c**) HbF levels and the recessive model for SNP rs4895441 in the HU+ group, **d**) HbF levels and the recessive model for SNP rs9402686 in the HU+ group, **e**) HbF levels and the dominant model for SNP rs11759553 in the HU+ group, **f**) HbF levels and the recessive model for SNP rs4895441 in males, **g**) HbF levels and the recessive model for SNP rs9402686 in males. Image edited using https://www.biorender.com

The results of a significant association were observed for rs7557939 under the overdominant model (*p* = 0.007), with a mean of 16.85 ± 7.96 for GG + AA compared with GA (7.12 ± 4.57), in the HU- group (Fig. 1a).

#### Criterion 3

Analysis of genetic models for SNPs indicates no statistically significant differences between sexes for the *BCL11A* polymorphisms evaluated.

### Comparative analysis of HbF values and SNPs in the *HBS1L-MYB* intergenic region

The evaluation was performed according to the criteria mentioned above. The data for the analysis of *HBS1L-MYB* intergenic region SNPs for each criterion is presented in Supplementary Table S3.

#### Criterion 1

A significant association was observed for rs11759553 under the dominant model (*p* = 0.025, Fig. 1.b), with higher HbF levels in the AT + TT genotypes (17.83 ± 7.77) compared with the AA genotype (12.25 ± 8.04).

#### Criterion 2

A significant association was identified in the HU+ group for rs4895441 under the recessive model (*p* = 0.032, Fig. 1c), with higher HbF levels in individuals with the minor allele (GG) (28.60 ± 0.92) compared to AA + AG carriers (16.45 ± 7.46). Similarly, rs9402686 showed a significant association under the recessive model (*p* = 0.040, Fig. 1d), with higher median HbF levels in individuals with the minor allele (AA) [28.65 (28.0–29.3)] compared to GG + GA carriers [16.45 (3.7–32.0)]. Additionally, rs11759553 was significantly associated with HbF levels under the dominant model in the same group (*p* = 0.015, Fig. 1e), with higher HbF levels observed in the presence of the minor allele (20.98 ± 7.06) compared to homozygous dominant individuals (AA; 13.68 ± 7.14).

#### Criterion 3

Significant associations were identified in male individuals for rs4895441 in the recessive model (*p* = 0.032, Fig. 1f), with higher median HbF levels in individuals with the minor allele (GG) [28.65 (28.0–29.3)] compared with those with the dominant allele [7.95 (2.0–29.0)]. Likewise, rs9402686 in males was also associated with HbF levels in the same model (*p* = 0.038, Fig. 1.g), with higher HbF values observed in individuals with the minor allele, AA [28.65 (28.0–29.3)], compared to GG + GA carriers [7.95 (2.0–29.0)].

### Variation in HbF levels by sex, HU use, and analyzed polymorphisms

Multiple linear regression models (with dummy variables) were fitted to assess the impact of the variables under investigation on HbF levels. The model proved significant when the variables “use of HU,” “sex,” “rs4895441,” “rs1427407” (considering the additive model for both polymorphisms), and “rs7557939” (recessive model) were included, and indicated that 46.5% of the variation in HbF levels is explained by the aforementioned predictor variables (R²: 0.465, F = 4.35, df1 = 7, df2 = 35, *p* = 0.001) (Table 3).

The analysis showed that HbF levels are significantly higher in: (i) females, compared to males; (ii) individuals who use HU; (iii) carriers of the TG and GG genotypes of the rs1427407 variant compared to homozygous T allele carriers; (iv) carriers of the GG genotype of the rs7557939 variant compared to individuals with AA and AG genotypes. On the other hand, homozygous carriers of the A allele of the rs4895441 variant have lower levels of HbF compared to carriers of the GG genotype (Table 3).

**Table 3:** Linear regression analysis to assess the influence of sex, HU use, and variants on HbF levels (*n* = 43)

		Overall Model Test							
Model	*R* ^2^	F		df 1	df2	*p*-value			
	0.465	4.35		7	35	0.001			
Preditor				95%CI					
	B^b^		β^β^	Range			p-value	VIF	Tolerance
Female-Male^a^	0.735		0.645	0.0992-1.191			**0.022**	1.1	0.911
HU^+^ - HU^− a^	0.966		0.848	0.2448–1.451			**0.007**	1.19	0.841
HBS1L-MYB rs4895441 (Minor allele: G ) - AA-GG^a^	-1.964		-1.724	-2.4867			**0.008**	1.1	0.913
	-1.367		-1.199	-2.7991			0.091		
*HBS1L-MYB rs4895441 (Minor allele: G) - *AG-GG^a^									
BCL11A rs1427407 (Minor allele: T) - TG-TT^a^	2.704		2.373	0.5265–4.219			**0.013**	1.07	0.934
*BCL11A rs1427407 (Minor allele: T)* - GG-TT^a^	2.692		2.362	0.5716–4.153			**0.011**		
*BCL11A rs7557939 (Minor allele: G)* - GG-AA + AG^a^	1.024		0.899	0.1518–1.646			**0.02**	1.17	0.853

^a^: reference level; ^b^: the magnitude of the effect (unstandardized regression coefficient – B and standardized regression coefficient – β) indicates the estimated change in HbF levels relative to the reference level; Multicollinearity (VIF < 10; tolerance > 0.2)

### Analysis of linkage disequilibrium and functionality of SNPs in the *BCL11A* gene and the *HBS1L-MYB* intergenic region

**Fig. 2:**
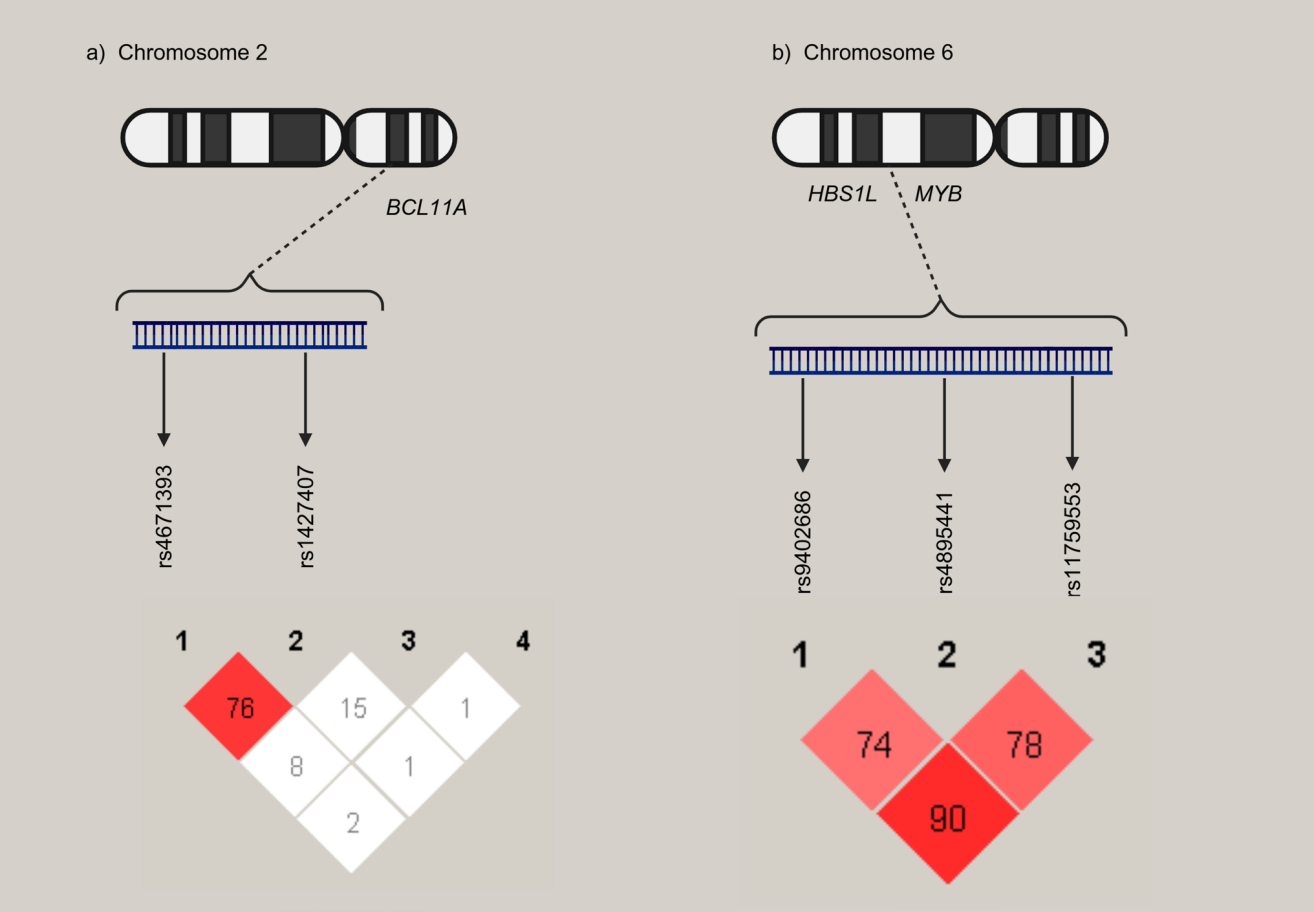
Block map for *BCL11A* SNPs and the *HBS1L-MYB* intergenic region. **A**) Proximity relationship between SNPs rs4671393 (1), rs1427407 (2), rs7557939 (3), and rs11886868 (4); **B**) relationship between SNPs rs9402686 (1), rs4895441 (2), and rs11759553 (3). The numbers within the blocks indicate the R^2^ for the analysis of variant pairs. The colors represent the degree of linkage disequilibrium: the more intense the red, the stronger the linkage disequilibrium. Image edited using https://www.biorender.com

The LD analysis showed that four pairs of variants had significant LD (Fig. 2). The SNP pairs rs4671393 and rs1427407 (*BCL11A*), as well as rs9402686, rs4895441, and rs11759553 (*HBS1L-MYB*), show strong LD.

Searches for location and functionality (Table 4) revealed that SNP rs4671393 is in a regulatory region of the genome that has been identified as a possible binding site for SMARCA4, a transcription factor, component of the SWI/SNF complex, involved in chromatin remodeling and transcriptional regulation, as evidenced by ChIP-seq data (RegulomeDB). The same SNP was identified in an active regulatory region in primary hematopoietic cells and B cells. Rs1427407 was also identified in primary hematopoietic cells and B cells. ChIP-seq analysis of this variant indicated an interaction with GATA-1, a transcription factor essential for the differentiation of erythroid and megakaryocyte cells. It is important to note that studies have shown that this region corresponds to an erythroid enhancer located in intron 2 of *BCL11A*, where its activity is modulated by genetic variations, primarily by rs1427407, which has been identified as a functional variant capable of altering the binding of erythroid transcription factors, impacting the expression of *BCL11A* and, consequently, HbF levels [35].

The rs9402686 showed a binding site for POLR2H in venous blood-derived cells, an essential subunit of RNA polymerase II. The rs4895441 was associated with CD8 + T cells and fetal thymus and showed ChIP-seq binding with CBFA2T3 and HMBOX1, both transcription factors involved in hematopoiesis and cell differentiation. The rs11759553 was identified in CD8 + T cells and fetal thymus, like rs4895441. The SNP also showed ChIP-seq binding to the same transcription factor identified in the analysis of rs4895441, CBFA2T3.

**Table 4:** Location and functionality data for SNPs in the *BCL11A* and *HBS1L-MYB* genes

SNP ID	POSITION	HaploReg	RegulomeDB
rs4671393	chr2:60493815–60,493,816	Primary hematopoietic cells / B cells	SMARCA4
rs1427407	chr2:60490907–60,490,908	Primary hematopoietic cells / B cells	GATA-1/Hematopoietic progenitor cell
rs9402686	chr6:135106678–135,106,679	Information unavailable	Venous blood
rs4895441	cr6:135105434–135,105,435	CD8 + T cell / Fetal thymus	CBFA2T3 and HMBOX1 / Venous blood
rs11759553	cr6:135101157–135,101,158	CD8 + T cell / Fetal thymus	CBFA2T3 / Venous blood

SNP: Single nucleotide polymorphism; eQTL: Expression of Quantitative Locus Trait

## Discussion

This study demonstrated associations between HU use, β^S^ haplotypes, and genetic variants in *BCL11A* and in the *HBS1L-MYB* region, which were associated with increased HbF levels, reinforcing that both genetic background and HU therapy contribute to interindividual variability in treatment response. Significant interactions were observed between HU therapy and β^S^ haplotypes, indicating that both pharmacological and genetic factors contribute to HbF variability in this population. These findings reinforce previous evidence regarding the role of HbF-modulating polymorphisms and provide additional data on their combined effects in a population from Bahia, Brazil.

In this study, individuals receiving HU had significantly higher HbF levels than untreated individuals, reinforcing HU as the primary pharmacological therapy for inducing HbF in patients with SCA. These results are consistent with previous research demonstrating that HU therapy significantly increases HbF levels in individuals with SCA. These findings are consistent with previous studies demonstrating significant increases in HbF following HU therapy in both adult and pediatric patients with SCA [36–38]. Although the exact mechanisms underlying HU-induced HbF production are not yet fully understood, these results reinforce the importance of treatment adherence and highlight the role of HU as a key disease-modifying agent [39], remaining the primary therapeutic strategy for increasing HbF levels and reducing disease severity, including vaso-occlusive crises and hospitalization rates [40].

It is also known that the β^S^ haplotype profile modulates HbF levels in SCA. In this study, the haplotype profile, sex, and HU use showed a statistically significant interaction. Male individuals carrying Bantu chromosomes who do not use HU have the lowest levels of HbF. The Bantu haplotype has been associated with more severe clinical conditions due to its link with low HbF levels and increased oxidative stress, inflammation, and DNA damage [41–43]. Supporting this observation, Santana et al., 2025 described a significant increase in levels of biochemical parameters, such as platelets, AST (aspartate aminotransferase), DB (direct bilirubin), IB (indirect bilirubin), and LDH (lactate dehydrogenase) in Bantu/Bantu individuals not using HU, compared to drug users with the same haplotype, among the same patients investigated in this study [22]. Elevated levels of these markers have been associated with vaso-occlusive processes, as well as with various clinical complications, such as thrombosis, pulmonary hypertension, leg ulcers, renal dysfunction, priapism, and an increased risk of death [44–48]. Taken together, these results underscore the critical interaction between haplotype profile and HU therapy in modulating HbF levels and disease severity.

Considering that HbF variability is multifactorial, we further evaluated whether biological sex could influence the interaction between β^S^ haplotypes and HU response. When sex was incorporated into the analysis, females presented significantly higher HbF levels than males, particularly among individuals receiving HU therapy. In this study, women with the Bantu/Bantu haplotype who received HU exhibited higher HbF levels than untreated males with the same haplotype profile. Di Mauro et al. demonstrated that males with SCD tend to exhibit a reduced response to HU therapy and an increased inflammatory burden compared to females, reinforcing the influence of biological sex on disease modulation and treatment response [49]. A large cohort study observed significantly higher HbF levels in females than in males, even among patients receiving HU therapy, supporting the hypothesis that sex-related mechanisms may influence HbF regulation [50]. These findings suggest that HbF modulation may result from a complex, multifactorial interaction rather than from the isolated effect of a single variable.

The available evidence indicates that sex effects on HbF levels operate independently of haplotype, with the X-linked F-cell production (FCP) *locus* as the dominant factor modulating haplotype effects on HbF expression [51]. The X-linked FCP *locus* accounts for approximately 40% of HbF variation in SCA, serving as a primary determinant of how other genetic factors, including β-globin haplotypes, influence final HbF levels [52]. However, direct haplotype-specific analyses of sex differences are limited in the literature. These findings should be interpreted with caution, as they may reflect the influence of the underlying genetic structure, including haplotype distribution, rather than a direct biological effect of sex. Additionally, recent data from Arcanjo et al. (2026), in a cohort of 409 patients with SCA in Brazil, demonstrated a higher frequency of severe clinical complications such as stroke, avascular necrosis, leg ulcers, priapism, and acute chest syndrome in male individuals [53]. These findings, together with the results of the present study, suggest that genetic and possibly therapeutic factors, such as HU use, may contribute to differences in the clinical presentation of the disease between the sexes.

The influence of polymorphisms on the genetic modulation of HbF levels has been extensively studied, particularly the impact of variants in the *BCL11A* gene and the *HBS1L-MYB* intergenic region [54–56]. In this study, we identified that carriers of alleles with variants in different polymorphisms in *BCL11A* and *HBS1L-MYB* may exhibit higher levels of HbF, which could affect the clinical condition of individuals with homozygous HbS.

Among the *BCL11A* SNPs, only significant associations involving rs7557939 were identified in untreated individuals (HU-), particularly under overdominant genetic models, with respect to criterion 2 of this study. A significantly greater effect on HbF levels was observed in the presence of the minor allele (GG) of the rs7557939 variant associated with the major allele (A) compared to AG genotypes (*p* = 0.007). Although this variant has not yet been widely highlighted for its association with increased HbF levels compared to other SNPs, the results from this population are consistent with studies by other authors that also observed increased HbF levels associated with the G allele of this variant in individuals with SCA [57].

Other studies have also linked the GG genotype to higher HbF levels, reinforcing the role of this genetic variant in increasing HbF [58]. These findings are biologically plausible given the central role of *BCL11A* in the silencing of γ-globin and the transition from fetal to adult hemoglobin [59]. Variants located in intronic erythroid enhancer regions of *BCL11A*, such as rs7557939, may alter transcription factor binding and erythroid-specific regulatory activity, directly influencing HbF expression [60].

In this context, the association observed for rs7557939 in individuals not using HU may indicate that this variant contributes to endogenous HbF production rather than exclusively to the pharmacological response. These findings reinforce the multifactorial nature of HbF regulation and underscore the importance of *BCL11A* variants as modulators of hematological variability in SCA.

GWAS studies have identified polymorphisms in the *HBS1L-MYB* intergenic region as the most relevant modifiers of HbF levels, with particular emphasis on the HMIP-2 block [61, 62] (references). In this study, individuals carrying allelic variants of *HBS1L-MYB*, analyzed under different criteria for the polymorphisms rs11759553, rs4895441, and rs9402686, showed significant variation in HbF levels, indicating that carriers of minor alleles for these variants have higher HbF levels (Supplementary Table S2).

In the *HBS1L-MYB* intergenic region, the rs11759553 variant showed statistically significant associations with increased HbF levels, especially in the presence of the minor allele (T). This finding is consistent with studies conducted in geographically close populations, suggesting shared genetic characteristics that may influence the progression of SCA in Brazil [63, 64]. Furthermore, this variant has been associated with improvements in hematological parameters, such as hematocrit and hemoglobin concentration, particularly in individuals receiving HU, thereby alleviating disease symptoms [65]. Thus, the results of this study reinforce the importance of this genetic factor in individuals with SCA, as it can both modulate the response to medication and mitigate disease progression.

The rs9402686 showed a significant association with HbF levels in this study. Some studies have shown that the same polymorphism, in a specific sample of Nigerian patients, was strongly associated with higher levels of HbF [66]. In the landmark Cooperative Study of Sickle Cell Disease (CSSCD) and Brazilian cohorts, *HBS1L-MYB* SNPs, including rs9402686, were strongly associated with HbF variation [67]. Carriers of the minor allele at rs9402686 (AA) exhibit improved clinical parameters, including a reduced reticulocyte count, higher peripheral oxygen saturation, and a lower risk of acute chest syndrome [68]. These clinical benefits appear to be mediated primarily by higher concentrations of HbF. The mechanism underlying these findings appears to be that the increased HbF at rs9402686 reduces transcription factor binding efficiency, thereby decreasing MYB expression and, consequently, increasing HbF production [69, 70].

In this study, we also found that users of hydroxyurea and carriers of the minor allele at rs4895441 have higher HbF levels. It is known that rs4895441, located in the HMIP-2 block, negatively regulates MYB [71]. It is well known that c-MYB is involved in regulating HbF production [72]. The MYB gene, which encodes the c-MYB transcription factor, plays an important role in regulating globin gene expression, cell cycle progression, and erythropoiesis [73, 74]. Additionally, c-MYB can activate repressor genes of γ-globin and inhibit HbF expression [75]. Interestingly, Cardoso and colleagues (2014), evaluating polymorphisms in HMIP-2 in individuals with SCA in northern Brazil, identified that among the variants rs2838451 (HMIP-1), rs4895441, and rs9399137 (HMIP-2), only rs4895441 was significantly associated with HbF levels [76]. Possibly, the relationship of this variant with c-MYB expression is the reason why individuals carrying the minor allele of rs4895441 exhibit higher HbF levels in response to HU use.

Although the individual identification of the relationship between *BCL11A* and *HBS1L-MYB* variants is significantly associated with HbF variation, in our analysis, sex and the use of hydroxyurea (HU) in conjunction with the rs1427407 and rs7557939 variants in BCL11A and rs4895441 in HBS1L-MYB are responsible for 46.5% of the variation in HbF levels, confirming the importance of these variables evaluated independently. The model indicates that females have higher HbF levels than males, and that HU use combined with inheritance of lower-allele variants significantly increases HbF levels in sickle cell anemia (SCA). Part of the HbF variability cannot be explained by the model, and this likely reflects interactions with other genes, epigenetic modifications, or variants of smaller effect [77]. Despite this, these findings reinforce the scope of considerations for the management, optimization, and clinical monitoring of SCA patients.

The presence of LD between the rs4671393 and rs1427407 variants of the *BCL11A* gene and the rs11759553, rs9402686, and rs4895441 variants of the *HBS1L-MYB* intergenic region suggests a possible functional interaction that may help regulate HbF production, thereby favoring its expression [78].

The variants in *BCL11A* in LD are in potentially regulatory regions associated with SMARCA4 and GATA-1 binding sites, which are essential for the regulation of hematopoietic cell production and differentiation, suggesting a possible functional role that requires experimental validation. SMARCA4, also known as BRG1, is an important transcription factor that acts in chromatin remodeling, exposing hematopoietic genes, and acting as a differential regulator of the self-renewal and renewal of hematopoietic stem cells, which was evidenced after its silencing by RNAi, which led to impaired self-renewal capacity of these cells [79].

In addition, it acts as a differential regulator of GATA-1 transcription, a key transcription factor in erythropoiesis that recruits co-regulatory complexes to drive gene activation and repression during the production of primary erythroid cells [80]. GATA-1 forms a complex with the DNA-binding transcription factors SCL/TAL1 and the structural proteins LMO2 and LDB1 to mediate the enhancer’s interaction with target promoters for gene activation [81, 82]. GATA-1 and LDB1 recruit the chromatin modifiers CBP/P300 and the BRG1 catalytic subunit of SWI/SNF to the β-globin locus control region (LCR) to promote erythroid differentiation and the formation of higher-order chromatin loops [88, 83, 84]. SMARCA4 (BRG1) and GATA-1, together with various factors, contribute to the regulation of LCR-γ-globin chromatin loop formation [85].

There was also evidence of linkage disequilibrium (LD) among the variants rs4895441, rs4902686, and rs11759553, which may also suggest that these SNPs are associated with the expression of genes essential for HbF production in the individuals who participated in this study [86]. Therefore, the LD patterns observed among these SNPs may reflect haplotype blocks that contribute to the regulation of HbF expression, offering potential targets for therapeutic interventions to increase HbF levels in patients with SCA.

All the *HBSL1-MYB* contributed to the increase in HbF under the three criteria analyses. The rs4895441 (involved with the regression model that explains 46.5% of the variation in HbF found) and rs11759553 are involved in key binding sites with transcription factors for hematopoietic cell production, such as CBFA2T3 (translocated alpha-2 subunit of the nuclear binding factor), also known as ETO2, a key transcriptional co-regulator in hematopoiesis and the regulation of differentiation of cell lines that contribute to the formation of blood components, such as erythrocytes and platelets [87]. The loss of ETO2 in murine and human erythroid progenitors results in delayed hemoglobin switching and the persistence of embryonic/fetal hemoglobin in adults, potentially representing a target for drugs used to treat β-hemoglobinopathies [88, 89]. To our knowledge, few studies have evaluated these haplotypic associations in Brazilian individuals with SCA.

From a clinical perspective, these findings suggest that the genetic profile of polymorphisms associated with HbF and β^S^ haplotypes may help predict individual response to HU therapy. These findings may, in the future, contribute to personalized medicine strategies, although validation in larger cohorts is necessary. Furthermore, identifying individuals with high-risk haplotypes may enable earlier interventions and improved clinical management.

## Conclusions

The study highlights the relevance of genetic factors in modulating HbF levels in patients with SCA. Significant associations were identified between specific SNPs, particularly rs4895441, rs1427407, and rs7557939, when considered in relation to sex and HU use, indicating that these variables together modulate HbF levels and account for approximately 46% of the variation in HbF. Analysis of genetic variants revealed LD patterns, suggesting functional interactions that favor the regulation of HbF production. From a clinical standpoint, identifying genetic variants associated with hydroxyurea response can help healthcare professionals better predict therapeutic outcomes and optimize treatment strategies for patients with sickle cell anemia.

## Supplementary Information

Below is the link to the electronic supplementary material.

Supplementary Material 1

## Data Availability

No datasets were generated or analysed during the current study.

## References

[1] Thomson A et al (2023) Global, regional, and national prevalence and mortality burden of sickle cell disease, 2000–2021: a systematic analysis from the Global Burden of Disease Study 2021. Lancet Haematol 10:585–599. 10.1016/S2352-3026(23)00118-7 10.1016/S2352-3026(23)00118-7 PMC10390339 37331373

[2] Kato GJ, Piel FB, Reid CD, Gaston MH, Ohene-Frempong K, Krishnamurti L, Smith WR, Panepinto JA, Weatherall DJ, Costa FF et al (2018) Sickle cell disease. Nat Reviews Disease Primers 4:18. 10.1038/nrdp.2018.10 10.1038/nrdp.2018.10 29542687

[3] Steinberg MH (1998) Pathophysiology of sickle cell disease. Baillière’s Clin Haematol 11:163–184. 10.1016/s0950-3536(98)80074-7 10872477 10.1016/s0950-3536(98)80074-7

[4] Musialek M, Rybaczek D (2021) Hydroxyurea-The Good, the Bad and the Ugly. Genes 12:1096–1104. 10.3390/genes12071096 34356112 10.3390/genes12071096 PMC8304116

[5] Dong M, Mcgann PT (2021) Changing the clinical paradigm of hydroxyurea treatment for sickle cell anemia through precision medicine. Clin Pharmacol Ther 109:73–81. 10.1002/cpt.2028 32869281 10.1002/cpt.2028 PMC7902468

[6] Bhanushali AA, Patra PK, Nair D, Verma H, Das BR (2015) Genetic variant in the *BCL11A* (rs1427407), but not *HBS1-MYB* (rs6934903) loci associate with fetal hemoglobin lev, els in Indian sickle cell disease patients. Blood Cells Molecules Dis 54:4–8. 10.1016/j.bcmd.2014.10.003 10.1016/j.bcmd.2014.10.003 25457385

[7] Allard P, Alapan Y, Nouraie M et al (2022) Genetic modifiers of fetal hemoglobin affect the course of sickle cell disease in patients treated with hydroxyurea. Haematologica 107(6):1234–1243. 10.3324/haematol.2021.278952 10.3324/haematol.2021.278952 PMC9244815 34706496

[8] Friedrisch JR, Sheehan VA et al The role of *BCL11A* and *HMIP-2* polymorphisms on endogenous and hydroxyurea-induced levels of fetal hemoglobin in sickle cell anemia patients from southern Brazil (2016). Blood Cells Mol Dis 62:32–37. 10.1016/j.bcmd.2016.11.002 10.1016/j.bcmd.2016.11.002 PMC5972079 27838552

[9] Pule GD, Mowla S, Novitzky N, Wiysonge CS, Wonkam A (2015) A systematic review of known mechanisms of hydroxyurea-induced fetal hemoglobin for treatment of sickle cell disease. Expert Rev Hematol 8(5):669–79. 10.1586/17474086.2015.1078235 10.1586/17474086.2015.1078235 PMC4829639 26327494

[10] Sankaran VG, Menne TF, Xu J, Akie TE, Lettre G, Handel BV, Mikkola HKA, Hirschhorn JN, Cantor AB, Orkin SH (2008) Human Fetal Hemoglobin Expression Is Regulated by the Developmental Stage-Specific Repressor *BCL11A*. Science 322:1839–1842. 10.1126/science.1165409 19056937 10.1126/science.1165409

[11] Trakarnsanga K, Wilson MC, Lau W, Singleton BK, Parsons SF, Sakuntanaga P, Kurita R, Nakamura Y, Anstee DJ, Frayne J (2014) Induction of adult levels of β-globin in human erythroid cells that intrinsically express embryonic or fetal globin by transduction with KLF1 and *BCL11A-XL*. Haematologica 99:1677–1685 10.3324/haematol.2014.110155 PMC4222483 25107887

[12] Sales RR, Nogueira BL, Tosatti JAG, Gomes KB, Luizon MR (2022) Do Genetic Polymorphisms Affect Fetal Hemoglobin (HbF) Levels in Patients With Sickle Cell Anemia Treated With Hydroxyurea? A Systematic Review and Pathway Analysis. Front Pharmacol 12:1. 10.3389/fphar.2021.779497 10.3389/fphar.2021.779497 PMC8814522 35126118

[13] Menzel S, Jiang J, Silver N, Gallagher J, Cunningham J, Surdulescu G, Lathrop M, Farrall M, Spector TD, Thein SL (2007) The *HBS1L-MYB* intergenic region on chromosome 6q23.3 influences erythrocyte, platelet, and monocyte counts in humans. Hematopoiesis 110:3624–3626. 10.1182/blood-2007-05-093419 10.1182/blood-2007-05-093419 17712044

[14] Ramsay RG, Gonda TJ (2008) MYB function in normal and cancer cells. Nat Rev Cancer 8:523–534. 10.1038/nrc2439 18574464 10.1038/nrc2439

[15] Alshaikh FS, Deifalla A, Sequeira RP, Woodman A (2025) Gene polymorphisms predicting response to hydroxyurea treatment in Bahraini patients with sickle cell disease. Expert Rev Hematol 18(11):999–1012. 10.1080/17474086.2025.2546575 10.1080/17474086.2025.2546575 40781956

[16] Rizo-de la Torre LC, Borrayo-López FJ, Perea-Díaz FJ, Aquino E, Venegas M, Hernández-Carbajal C, Espinoza-Mata LL, Ibarra-Cortés B (2022) Fetal hemoglobin regulating genetic variants identified in homozygous (HbSS) and heterozygous (HbSA) subjects from South Mexico. J Trop Pediatr 68:fmac073. 10.1093/tropej/fmac073 36130307 10.1093/tropej/fmac073

[17] Akbulut-Jeradi N, Fernandez MJ, Al Khaldi R, Sukumaran J, Adekile A (2021) Unique polymorphisms at BCL11A, HBS1L-MYB and HBB loci associated with HbF in Kuwaiti patients with sickle cell disease. J Pers Med [Internet] 11(6):567. Available from: 10.3390/jpm11060567 10.3390/jpm11060567 PMC8234980 34204365

[18] Cardoso GL, Diniz IG, Silva ANLM, Cunha DA, Silva Junior JS, Uchôa CTC, Santos SEB, Trindade SMS, Cardoso MSO, Guerreiro JF (2014) DNA polymorphisms at BCL11A, HBS1L-MYB and Xmn1-HBG2 site loci associated with fetal hemoglobin levels in sickle cell anemia patients from Northern Brazil. Blood Cells Molecules Dis 53:176–179. 10.1016/j.bcmd.2014.07.006 10.1016/j.bcmd.2014.07.006 25084696

[19] Fanis P, Kousiappa I, Phylactides M, Kleanthous M (2014) Genotyping of *BCL11A* and *HBS1L*-*MYB* SNPs associated with fetal haemoglobin levels: a SNaPshot minisequencing approach. BMC Genomics 15:108. 10.1186/1471-2164-15-108 24502199 10.1186/1471-2164-15-108 PMC3922441

[20] Banan M, Bayat H, Azarkeivan A, Mohammadparast S, Kamali K, Farashi S, Bayat N, Khani MH, Neishabury M, Najmabadi H (2012) The XmnI and *BCL11A* single nucleotide polymorphisms may help predict hydroxyurea response in Iranian β-thalassemia patients. Hemoglobin 36:371–380. 10.3109/03630269.2012.691147 22686296 10.3109/03630269.2012.691147

[21] Saiki RK, Scharf S, Faloona F, Mullis KB, Horn GT, Erlich HÁ, Arnheim N (1985) Enzymatic amplification of beta-globin genomic sequences and restriction site analysis for diagnosis of sickle cell anemia. Science 230(4732):1350–1354. 10.1126/science.2999980 2999980 10.1126/science.2999980

[22] Santana ILR, Magalhães R, Bernardo VS, Magalhães Filho MF, Silva PLP, Venancio LPR (2025) β^S^ haplotypes: Genetic profile and association with biochemical parameters in individuals with sickle cell anemia in Western Bahia, Brazil. Hum Gene 45:201438. 10.1016/j.humgen.2025.201438

[23] Sutton M, Bouhassira EE, Nagel RL (1989) Polymerase chain reaction amplification applied to the determination of beta-like globin gene cluster haplotypes. Am J Hematol 32:66–69. 10.1002/ajh.2830320113 2757004 10.1002/ajh.2830320113

[24] Okumura JV, Silva DGH, Torres LS, Belini-Junior E, Venancio LPR, Carrocini GCS et al (2019) Atypical β-S haplotypes: classification and genetic modulation in patients with sickle cell anemia. J Hum Genet 64(3):239–48. https://www.nature.com/articles/s10038-018-0554-4 10.1038/s10038-018-0554-4 30622282

[25] Collins A, Ke X (2012) Primer1: Primer Design Web Service for Tetra-Primer ARMS-PCR. Open Bioinf J 6:55–58. 10.2174/1875036201206010055

[26] Fanis P, Kousiappa I, Phylactides M, Kleanthous M (2018) Quantitative trait loci influencing Hb F levels in Southern Thai Hb E (HBB: c.79G > A) heterozygotes. Int J Hemoglobin Res 6:15108. 10.1186/1471-2164-15-108

[27] Clarke GM, Anderson CA, Pettersson FH, Cardon LR, Morris AP, Zondervan KT (2011) Basic statistical analysis in genetic case-control studies. Nat Protoc 6(2):121–33. Available from: 10.1038/nprot.2010.182 10.1038/nprot.2010.182 PMC3154648 21293453

[28] Kim Y, Chi Y-Y, Zou F (2020) An efficient integrative resampling method for gene-trait association analysis. Genet Epidemiol [Internet] 44(2):197–207. Available from: 10.1002/gepi.22271 10.1002/gepi.22271 31820489

[29] Liu H-M, Zheng J-P, Yang D, Liu Z-F, Li Z, Hu Z-Z et al (2021) Recessive/dominant model: Alternative choice in case-control-based genome-wide association studies. PLoS One 16(7):e0254947. Available from: 10.1371/journal.pone.0254947 10.1371/journal.pone.0254947 PMC8294554 34288964

[30] Wall JD, Pritchard JK (2003) Haplotype blocks and linkage disequilibrium in the human genome. Nat Rev Genet 4(8):587–597. 10.1038/nrg1123 12897771 10.1038/nrg1123

[31] Ofakunrin AOD, Oguche S, Adekola K, Okpe ES, Afolaranmi TO, Diaku-Akinwumi IN et al (2020) Effectiveness and safety of hydroxyurea in the treatment of sickle cell anaemia children in Jos, north central Nigeria. J Trop Pediatr [Internet] 66(3):290–298. 10.1093/tropej/fmz070 31608959 10.1093/tropej/fmz070 PMC7249733

[32] Mohammad SNNA, Iberahim S, Wan Ab Rahman WS, Hassan MN, Edinur HA, Azlan M et al (2022) Single nucleotide polymorphisms in XMN1-HBG2, HBS1L-MYB, and BCL11A and their relation to high fetal hemoglobin levels that alleviate anemia. Diagnostics 12:1–13. 10.3390/diagnostic 10.3390/diagnostics12061374 PMC9221560 35741184

[33] Lettre G, Sankaran VG, Bezerra MAC, Araujo AS, Uda M, Sanna S et al (2008) DNA polymorphisms at the *BCL11A, HBS1L-MYB*, and β-globin loci associate with fetal hemoglobin levels and pain crises in sickle cell disease. Proc Natl Acad Sci USA 105:11869–11874. 10.1073/pnas08047 99105 18667698 10.1073/pnas.0804799105 PMC2491485

[34] Huang T, Jiang H, Tang G, Li J, Huang X, Huang Z et al (2024) Efficacy and safety of hydroxyurea therapy on patients with β-thalassemia: a systematic review and meta-analysis. Front Med (Lausanne) [Internet] 11:1480831. 10.3389/fmed.2024.1480831 39882530 10.3389/fmed.2024.1480831 PMC11774989

[35] Bauer DE, Kamran SC, Orkin SH (2013) An erythroid enhancer of *BCL11A* subject to genetic variation determines fetal hemoglobin level. Science 342:253–257. 10.1126/science.1242088 24115442 10.1126/science.1242088 PMC4018826

[36] Kabuyi PL, Mbayabo G, Ngole M, Zola AL, Race V, Matthijs G, Geet CV, Tshilobo PL, Devriendt K, Mikobi TM (2023) Hydroxyurea treatment for adult sickle cell anemia patients in Kinshasa. EJHaem 4:3:595–601. 10.1002/jha2.735 37601858 10.1002/jha2.735 PMC10435708

[37] Kargutkar N, Sawant-Mulay M, Hariharan P, Chandrakala S, Nadkarni A (2023) Role of microRNA in hydroxyurea mediated HbF induction in sickle cell anaemia patients. Sci Rep 13:1 36611033 10.1038/s41598-022-25444-3 PMC9825386

[38] Santos B, Ginete C, Gonçalves E, Delgadinho M, Miranda A, Faustino P, Arez AP, Brito M (2024) Characterization of a cohort of Angolan children with sickle cell anemia treated with hydroxyurea. Blood Cells Mol Dis 105:102822102822. 10.1016/j.bcmd.2023.102822 10.1016/j.bcmd.2023.102822 38215581

[39] Silva-Pinto AC, Angulo IL, Brunetta DM, Neves FIR, Bassi SC, Santis GCD, Covas DT (2013) Clinical and hematological effects of hydroxyurea therapy in sickle cell patients: a single-center experience in Brazil. Sao Paulo Med J 131:4:238–243. 10.1590/1516-3180.2013.1314467 24141294 10.1590/1516-3180.2013.1314467 PMC10871833

[40] Quinn CT, Ware RE (2025) The modern use of hydroxyurea for children with sickle cell anemia. Haematologica. 10.3324/haematol.2023.284633 39781621 10.3324/haematol.2023.284633 PMC12050929

[41] Steinberg MH, Lu Z-H, Barton FB, Terrin ML, Charache S, Dover GJ et al (1997) Fetal hemoglobin in sickle cell anemia: Determinants of response to Hydroxyurea. Blood 89(3):1078–1088. 10.1182/blood.v89.3.1078 9028341

[42] da Silva Rocha LB, Dias Elias DB, Barbosa MC, Bandeira ICJ, Gonçalves RP (2012) DNA damage in leukocytes of sickle cell anemia patients is associated with hydroxyurea therapy and with HBB*S haplotype. Mutat Res 749(1–2):48–52. 10.1016/j.mrgentox.2012.08.003 22918118 10.1016/j.mrgentox.2012.08.003

[43] Bandeira ICJ, Rocha LBS, Barbosa MC, Elias DBD, Querioz JAN, Freitas MVC et al (2014) Chronic inflammatory state in sickle cell anemia patients is associated with HBB(*)S haplotype. Cytokine 65(2):217–221. 10.1016/j.cyto.2013.10.009 24290434 10.1016/j.cyto.2013.10.009

[44] Belcher JD, Mahaseth H, Welch TE, Vilback AE, Sonbol KM, Kalambur VS et al (2005) Critical role of endothelial cell activation in hypoxia-induced vasoocclusion in transgenic sickle mice. Am J Physiol Heart Circ Physiol 288(6):H2715–H2725. 10.1152/ajpheart.00986.2004 15665055 10.1152/ajpheart.00986.2004

[45] Stankovic Stojanovic K, Lionnet F (2016) Lactate dehydrogenase in sickle cell disease. Clin Chim Acta 458:99–102. 10.1016/j.cca.2016.04.035 27138446 10.1016/j.cca.2016.04.035

[46] Kato GJ, McGowan V, Machado RF, Little JA, Taylor J 6th, Morris CR et al (2006) Lactate dehydrogenase as a biomarker of hemolysis-associated nitric oxide resistance, priapism, leg ulceration, pulmonary hypertension, and death in patients with sickle cell disease. Blood 107(6):2279–2285. 10.1182/blood-2005-06-2373 16291595 10.1182/blood-2005-06-2373 PMC1895723

[47] Gladwin MT, Sachdev V, Jison ML, Shizukuda Y, Plehn JF, Minter K et al (2004) Pulmonary hypertension as a risk factor for death in patients with sickle cell disease. N Engl J Med 350(9):886–895. 10.1056/NEJMoa035477 14985486 10.1056/NEJMoa035477

[48] Nouraie M, Lee JS, Zhang Y, Kanias T, Zhao X, Xiong Z et al (2013) The relationship between the severity of hemolysis, clinical manifestations and risk of death in 415 patients with sickle cell anemia in the US and Europe. Haematologica 98(3):464–472. 10.3324/haematol.2012.068965 22983573 10.3324/haematol.2012.068965 PMC3659937

[49] Di Mauro M, El Hoss S, Nardo-Marino A, Stuart-Smith S, Strouboulis J, Gibson JS et al (2023) Males with sickle cell disease have higher risks of cerebrovascular disease, increased inflammation, and a reduced response to hydroxyurea. Am J Hematol 98(11):E341–E344. 10.1002/ajh.27074 37646569 10.1002/ajh.27074

[50] Balachandran N, Jella S, Aston H, Boghani F, Gazza C, Hazenberg E et al (2023) Sex-related differences in fetal hemoglobin levels in sickle cell patients. Blood 142:5304–5304. 10.1182/blood-2023-190193

[51] Chang YC, Smith KD, Moore RD, Serjeant GR, Dover GJ (1995) An analysis of fetal hemoglobin variation in sickle cell disease: the relative contributions of the X-linked factor, beta-globin haplotypes, alpha-globin gene number, gender, and age. Blood 85(4):1111–1117. 10.1182/blood.v85.4.1111.bloodjournal8541111 7531513

[52] Chang YP, Maier-Redelsperger M, Smith KD, Contu L, Ducroco R, de Montalembert M et al (1997) The relative importance of the X-linked FCP locus and beta-globin haplotypes in determining hemoglobin F levels: a study of SS patients homozygous for beta S haplotypes. Br J Haematol 96(4):806–814. 10.1046/j.1365-2141.1997.d01-2094 9074425 10.1046/j.1365-2141.1997.d01-2094.x

[53] Sales RR, Belisario AR, Faria G, Mendes F, Luizon MR, Viana MB (2020) Functional polymorphisms of BCL11A and HBS1L-MYB genes affect both fetal hemoglobin level and clinical outcomes in a cohort of children with sickle cell anemia. Ann Hematol 99:1453–1463 32447424 10.1007/s00277-020-04079-2

[54] Upadhye D, Jain D, Trivedi Y, Nadkarni A, Ghosh K, Colah R (2016) Influence of single nucleotide polymorphisms in the BCL11A and HBS1L-MYB gene on the HbF levels and clinical severity of sickle cell anaemia patients. Ann Hematol 95:1201–1203 27098811 10.1007/s00277-016-2675-1

[55] Chaouch L, Moumni I, Ouragini H, Darragi I, Kalai M, Chaouachi D et al (2016) rs11886868 and rs4671393 of BCL11A associated with HbF level variation and modulate clinical events among sickle cell anemia patients. Hematol (United Kingdom) 21:425–429 10.1080/10245332.2015.1107275 27077760

[56] Arcanjo GS, Silva AP, Diniz MV, Domingos IF, Pereira-Martins DA, Araújo AB et al (2026) A genetic risk score based on *BCL11A* and *HBS1L-MYB* variants predicts clinical severity in Brazilian sickle cell anaemia patients. Br J Haematol. 10.1111/bjh.70489. (bjh.70489) 41989145 10.1111/bjh.70489 PMC13267477

[57] Green NS, Ender KL, Pashankar F, Driscoll C, Giardina PJ, Mullen CA et al (2013) Candidate sequence variants and fetal hemoglobin in children with sickle cell disease treated with hydroxyurea. PLoS ONE 8(2):e55709. 10.1371/journal.pone.0055709 23409025 10.1371/journal.pone.0055709 PMC3567082

[58] Delgadinho M, Ginete C, Santos B, Miranda A, Brito M (2021) Genotypic Diversity among Angolan Children with Sickle Cell Anemia. Int J Environ Res Public Health 18(10):5417. 10.3390/ijerph18105417 34069401 10.3390/ijerph18105417 PMC8158763

[59] Bauer DE, Kamran SC, Lessard S, Xu J, Fujiwara Y, Lin C et al (2013) An erythroid enhancer of BCL11A subject to genetic variation determines fetal hemoglobin level. Science 342(6155):253–257. 10.1126/science.1242088 24115442 10.1126/science.1242088 PMC4018826

[60] Stadhouders R, Aktuna S, Thongjuea S, Aghajanirefah A, Pourfarzad F, Van IJcken W et al (2014) HBS1L-MYB intergenic variants modulate fetal hemoglobin via long-range MYB enhancers. J Clin Invest 124:1699–1710 24614105 10.1172/JCI71520 PMC3973089

[61] Thein SL, Menzel S, Peng X, Best S, Jiang J, Close J et al (2007) Intergenic variants of HBS1L-MYB are responsible for a major quantitative trait locus on chromosome 6q23 influencing fetal hemoglobin levels in adults. Proc Natl Acad Sci USA 104:11346–11351 17592125 10.1073/pnas.0611393104 PMC2040901

[62] Sankaran VG, Menne TF, Xu J, Akie TE, Lettre G, Van Handel B et al (2008) Human fetal hemoglobin expression is regulated by the developmental stage-specific repressor BCL11A. Science 322(5909):1839–1842. 10.1126/science.1165409 19056937 10.1126/science.1165409

[63] Silva WS, Lopes TS, Reis DS, Barreto DPS, Silva GS, Oliveira TWS, Lucena RCS, Baptista AF (2022) Aspectos sociodemográficos e clínicos de pacientes com doenças falciformes dos centros de referência em Salvador, Bahia. Brazilian J Health Rev 5:3. 10.34119/bjhrv5n3-215

[64] Aleluia MM, Santiago RP, Guarda CC, Fonseca TCC, Neves FI, Quinto RS, Figueiredo CVB, Yahouédéhou SCMA, Oliveira RM, Ferreira JRD (2017) Genetic modulation of fetal hemoglobin in hydroxyurea-treated sickle cell anemia. Am J Hematol 92:5:70–72. 10.1002/ajh.24680 10.1002/ajh.24680 PMC5389903 28195442

[65] Adeyemo TA, Ojewunmi OO, Oyetunji IA, Rooks H, Rees DC, Akinsulie AO, Akanmu AS, Thein SL, Menzel S (2018) A survey of genetic fetal-haemoglobin modifiers in Nigerian patients with sickle cell anaemia. PLoS ONE 13:6197927. 10.1371/journal.pone.0197927 10.1371/journal.pone.0197927 PMC5991720 29879141

[66] Lettre G, Sankaran VG, Bezerra MAC, Araújo AS, Uda M, Sanna S, Cao A, Schlessinger D, Costa FF, Hirschhorn JN (2008) DNA polymorphisms at the *BCL11A*, *HBS1L-MYB*, and β- globin loci associate with fetal hemoglobin levels and pain crises in sickle cell disease. Proceedings of the National Academy of Sciences of the United States of America 105:33:11869–11874. 10.1073/pnas.0804799105 10.1073/pnas.0804799105 PMC2491485 18667698

[67] Sales RR, Belisário AR, Faria G, Mendes F, Luizon MR, Viana MB (2020) Functional polymorphisms of *BCL11A* and *HBS1L-MYB* genes affect both fetal hemoglobin level and clinical outcomes in a cohort of children with sickle cell anemia. Ann Hematol 99(7):1453–1463. 10.1007/s00277-020-04079-2 32447424 10.1007/s00277-020-04079-2

[68] Stadhouders R, Aktuna S, Thongjuea S, Aghajanirefah A, Pourfarzad F, van Ijcken W et al (2014) *HBS1L-MYB* intergenic variants modulate fetal hemoglobin via long-range MYB enhancers. J Clin Invest 124(4):1699–1710. 10.1172/JCI71520 24614105 10.1172/JCI71520 PMC3973089

[69] Weatherall DJ (2001) Phenotype-genotype relationships in monogenic disease: lessons from the thalassaemias. Nat Rev Genet 2(4):245–255 11283697 10.1038/35066048

[70] Weatherall DJ (2001) Phenotype-genotype relationships in monogenic disease: lessons from the thalassaemias. Nat Rev Genet 2(4):245–255 11283697 10.1038/35066048

[71] Ramsay RG, Gonda RS (2008) MYB function in normal and cancer cells. Nat Rev Cancer 8(7):523–534 18574464 10.1038/nrc2439

[72] Cheung VS, Spielman RS (2002) The genetics of variation in gene expression. Nat Genet 32(Suppl):522–525 12454648 10.1038/ng1036

[73] Stadhouders R et al (2014) HBS1L-MYB intergenic variants modulate fetal hemoglobin via long-range MYB enhancers. J Clin Invest 124(4):1699–1710 24614105 10.1172/JCI71520 PMC3973089

[74] Cardoso GL et al (2014) DNA polymorphisms at *BCL11A, HBS1L-MYB* and Xmn1-HBG2 site loci associated with fetal hemoglobin levels in sickle cell anemia patients from Northern Brazil. Blood Cells Molecules Disease 53:176–179 10.1016/j.bcmd.2014.07.006 25084696

[75] Milton JN et al (2014) Prediction of fetal hemoglobin in sickle cell anemia using an ensemble of genetic risk prediction models. Circ Cardiovasc Genet 7:110–115 24585758 10.1161/CIRCGENETICS.113.000387 PMC3994553

[76] Sebastiani P, Farrell JJ, Alsultan A, Wang S, Edward HL, Shappell H et al (2015) BCL11A enhancer haplotypes and fetal hemoglobin in sickle cell anemia. Blood Cells Mol Dis 54(3):224–230. 10.1016/j.bcmd.2015.01.001 25703683 10.1016/j.bcmd.2015.01.001 PMC4341902

[77] Aleluia MM, Santiago RP, Guarda CC, Fonseca TCC, Neves FI, Quinto RZ, Figueiredo CVB, Yahouédéhou SCMA, Oliveira RM, Ferreira JRD et al (2017) Genetic modulation of fetal hemoglobin in hydroxyurea-treated sickle cell anemia. Am J Hematol 92:5:70–72. 10.1002/ajh.24680 10.1002/ajh.24680 PMC5389903 28195442

[78] Sales RR, Nogueira BL, Belisário AR, Mendes F, Faria G, Viana MB, Luizon MR (2021) Haplótipos de *BCL11A* e *HBS1L-MYB* associados com concentração elevada de hemoglobina fetal e interação entre estes loci na predição de desfechos clínicos e hematológicos em crianças com anemia falciforme. Hematology, transfusion and cell therapy 43:286–287

[79] Ropero P, Peral M, Sánchez-Martínez LJ, Rochas S, Gómez-Álvarez M, Nieto JM et al (2025) Phenotype of sickle cell disease. Correlation of haplotypes and polymorphisms in cluster β, *BCL11A*, and *HBS1L-MYB*. Pilot study. Front Med (Lausanne) 12:1347026. 10.3389/fmed.2025.1347026 10.3389/fmed.2025.1347026 PMC11864215 40012971

[80] Güneş C, Ramos-Merino L, Wu Y, Wangensteen KJ, Zaehres H, Araúzo-Bravo MJ, Heimann P, Bryja V, Göke J, Chang T et al (2019) Comparative RNAi Screens in Isogenic Human Stem Cells Reveal SMARCA4 as a Differential Regulator. Stem Cell Rep 12(5):1084–1098. 10.1016/j.stemcr.2019.03.012 10.1016/j.stemcr.2019.03.012 PMC6523874 31031192

[81] Țichil I, Mitre I, Zdrenghea MT, Bojan AS, Tomuleasa CI, Cenariu D (2024) A review of key regulators of steady-state and ineffective erythropoiesis. J Clin Med 13(9):2585. 10.3390/jcm13092585 10.3390/jcm13092585 PMC11084473 38731114

[82] Li L, Freudenberg J, Cui K, Dale R, Song S-H, Dean A, Zhao K, Jothi R, Love PE (2013) Ldb1-nucleated transcription complexes function as primary mediators of global erythroid gene activation. Blood 121(22):4575–4585. 10.1182/blood-2013-01-479451 23610375 10.1182/blood-2013-01-479451 PMC3668490

[83] Krivega I, Dale RK, Dean A (2014) Role of LDB1 in the transition from chromatin looping to transcription activation. Genes Dev 28(12):1278–1290. 10.1101/gad.239749.114 24874989 10.1101/gad.239749.114 PMC4066399

[84] Bultman SJ, Gebuhr TC, Magnuson T (2005) A Brg1 mutation that uncouples ATPase activity from chromatin remodeling reveals an essential role for SWI/SNF-related complexes in beta- globin expression and erythroid development. Genes Dev 19(23):2849–2861. 10.1101/gad.1364105 16287714 10.1101/gad.1364105 PMC1315392

[85] Letting DL, Rakowski C, Weiss MJ, Blobel GA (2003) Formation of a tissue-specific histone acetylation pattern by the hematopoietic transcription factor GATA-1. Mol Cell Biol 23(4):1334–1340. 10.1128/MCB.23.4.1334-1340.2003 12556492 10.1128/MCB.23.4.1334-1340.2003 PMC141148

[86] Guo X, Zhao Y, Kim J, Dean A (2022) Hemogen/BRG1 cooperativity modulates promoter and enhancer activation during erythropoiesis. Blood 139(24):3532–3545. 10.1182/blood.2021014308 35297980 10.1182/blood.2021014308 PMC9203704

[87] Sales RR, Nogueira BL, Belisário AR, Faria G, Mendes F, Viana MB, Luizon MR (2022) Fetal hemoglobin-boosting haplotypes of *BCL11A* gene and *HBS1L-MYB* intergenic region in the prediction of clinical and hematological outcomes in a cohort of children with sickle cell anemia. J Hum Genet 67:12:701–709. 10.1038/s10038-022-01079-0 36167770 10.1038/s10038-022-01079-0

[88] Steinauer N, Guo C, Huang C, Wong M, Tu Y, Freter CE, Zhang J (2019) Myeloid translocation gene CBFA2T3 directs a relapse gene program and determines patient-specific outcomes in AML. Blood Adv 3:9:1379–1393. 10.1182/bloodadvances.2018028514 31040112 10.1182/bloodadvances.2018028514 PMC6517668

[89] Kiefer CM, Lee J, Hou C, Dale RK, Lee YT, Meier ER, Miller JL, Dean A (2020) Embryonic erythropoiesis and hemoglobin switching require transcriptional repressor ETO2 to modulate chromatin organization. Nucleic Acids Res 48(16):9029–9043. 10.1093/nar/gkaa736 10.1093/nar/gkaa736 PMC7544236 32960220

