# Supplementary material for: BCL11A and HBS1L-MYB polymorphisms, in association with hydroxyurea and sex, modulate fetal hemoglobin levels in individuals with sickle cell anemia in the western region of Bahia, Brazil
Source: Mol Biol Rep. 2026 Jun 23;53(1):976. doi: 10.1007/s11033-026-12151-9 (PMC13290901; doi:10.1007/s11033-026-12151-9)
Supplement: Supplementary file 1 — Supplementary Material 1 [file 11033_2026_12151_MOESM1_ESM.docx]

**Table S1** – Sequence of primers used in the genotyping of rs11886868

| Sequence of primer oligonucleotides | |
| --- | --- |
| Forward outer | 5’ GTG GAG AGG TTT CTA TTC GGA ATA GGA T 3’ |
| Reverse outer | 5’TGA GGA GAC CCA AAC AGT TAA AGG TTA C 3’ |
| Forward inner | 5’AAT CCC AGA ATC ATT CTG CTC TGA GG 3’ |
| Reverse inner | 5’AGG ATA TCG TCT TTT GTG TTT AAT TTC ATG 3’ |

The table shows the sequence of primers used in the rs11886868 genotype. The first column indicates the type of primer, and the second column shows the oligonucleotide sequences for each primer.

**Table S2** – Analysis of *BCL11A* gene SNPs for each of the criteria.

| Criteria | SNP | n | Dominance model | | Median (Min–Max)*  Mean±SD of %HbF | p value | p* |
| --- | --- | --- | --- | --- | --- | --- | --- |
| 1 | rs4671393  (Minor allele: A) | GG – 22  GA – 19  AA – 2 | Additive model | GG *vs* GA *vs* AA | GG – 16.1±8.80  GA – 14.3±7.99  AA – 11.3±7.50 | 0.648^d^ |  |
|  |  |  | Dominant model | GG *vs* GA+AA | GG – 16.11±8.80  GA + AA – 14.06±7.82 | 0.424^b^ |  |
|  |  |  | Recessive model | AA *vs* GG+GA | AA – 11.3 (6.0 – 16.6)*  GG + GA – 13.7 (2.0 – 32.0)* | 0.513^a^ |  |
|  |  |  | Allelic model | A *vs* G | A – 14.90 (2-28)*  G – 13.70 (2 – 32)* | 0.608^a^ |  |
|  |  |  | Overdominant model | GA *vs* GG+AA | GA – 14.35±7.99  GG + AA – 16.19±8.51 | 0.60^b^ |  |
|  | rs1427407  (Minor allele: T) | GG - 21  GT – 21  TT - 1 | Additive model | GG *vs* GT | GG – 16.34±8.53  GT – 14.30±8.10  TT – 6.0% | 0.432^b^ |  |
|  |  |  | Dominant model | GG *vs* GT+TT | GG – 13.5 (4.50 – 32.0)*  GT+TT – 15.1 (2.0 – 28.0)* | 0.375^a^ |  |
|  |  |  | Allelic model | G *vs* T | G – 15.32±8.27  T – 13.93±8.10 | 0.521^b^ |  |
|  |  |  | Recessive model | **NE** |  |  |  |
|  |  |  | Overdominant model | GT *vs* GG+TT | GT – 15.30 (2.0 – 28.0)*  GG+TT – 12.80 (4.50 – 32.0)* | 0.601^a^ |  |
|  | rs7557939  (Minor allele: G) | AA – 10  AG – 25  GG – 8 | Additive model | GG *vs* AG *vs* AA | GG – 15.0 (6.0 – 29.50)*  AG – 12.1 (2.0 – 29.30)*  AA – 20.0 (7.40 – 32.0)* | 0.298^a^ |  |
|  |  |  | Dominant model | AA *vs* GA + GG | AA – 20.0 (7.40 – 32.0)*  GA + GG – 13.5 (2.0 – 29.5)* | 0.167^a^ |  |
|  |  |  | Recessive model | GG *vs* GA + AA | GG – 15.39±7.35  GA + AA – 15.04±8.60 | 0.917^b^ |  |
|  |  |  | Allelic model | G *vs* A | G – 14.09±8.19  A – 15.04±8.60 | 0.643^b^ |  |
|  |  |  | Overdominant model | GA *vs* GG + AA | GA – 12.1 (2.0 – 29.3)*  GG + AA – 16.8 (6.0 – 32.0)* | 0.157^b^ |  |
|  | rs11886868  (Minor allele: C) | TT – 30  TC – 10  CC – 3 | Additive model | TT *vs* TC *vs* CC | TT – 15.03±8.46  TC – 16.99±8.63  CC – 9.60±3.17 | 0.409^d^ |  |
|  |  |  | Dominant model | TT *vs* TC + CC | TT – 9.60±3.17  TC + CC – 15.52±8.44 | 0.238^b^ |  |
|  |  |  | Recessive model | CC *vs* TT + TC | CC – 15.03±8.46  TT + TC – 15.28±8.25 | 0.928^b^ |  |
|  |  |  | Allelic model | C *vs* T | C – 15.52±8.44  T – 15.28±8.25 | 0.930^b^ |  |
|  |  |  | Overdominant model | TC *vs* CC + TT | TC – 14.4 (7.70 – 32.0)*  CC + TT – 13.7 (2.0 – 29.5)* | 0.396^a^ |  |
| 2 (HU-) | rs4671393  (Minor allele: A) | GG - 8  GA - 9  AA - 0 | Additive model | GA *vs* GG | GG – 10.6±8.3  GA – 12.9±7.91 | 0.570^b^ |  |
|  |  |  | Allelic model | A *vs* G | A – 10.62±8.29  G – 11.70±7.95 | 0.749^b^ |  |
|  |  |  | Recessive model | **NE** |  |  |  |
|  |  |  | Dominant model | **NE** |  |  |  |
|  |  |  | Overdominant model | **NE** |  |  |  |
|  | rs1427407  (Minor allele: T) | GG – 8  GT – 9  TT – 0 | Additive model | GG *vs* GT | GG – 12.91±7.91  TG – 10.62±8.29 | 0.570^b^ |  |
|  |  |  | Allelic model | T *vs* G | T – 10.62±8,29  G – 11.70±7.95 | 0.749^b^ |  |
|  |  |  | Recessive model | **NE** |  |  |  |
|  |  |  | Dominant model | **NE** |  |  |  |
|  |  |  | Overdominant model | **NE** |  |  |  |
|  | rs7557939  (Minor allele: G) | AA – 3  AG – 9  GG - 4 | Additive model | AA *vs* AG *vs* GG | AA – 22.0 (7.4 – 24.6)*  AG – 5.9 (2.0 – 15.3)*  GG – 17.7 (8.2 – 29.5)* | **0.029^c^** | AA *vs* AG – 0.127  AA *vs* GG – 0.980  AG *vs* GG – 0.060 |
|  |  |  | Dominant model | AA *vs* GA + GG | AA – 18.0±9.27  GA + GG -10.35±7.31 | 0.135^b^ |  |
|  |  |  | Recessive model | GG *vs* GA + AA | GG – 16.16±8.14  GA + AA – 9.84±8.14 | 0.140^b^ |  |
|  |  |  | Allelic model | A *vs* G | A – 9.84±7.42  G – 10.35±7.31 | 0.862**^b^** |  |
|  |  |  | Overdominant model | GA *vs* GG + AA | GA – 7.12±4.57  GG + AA – 16.85±7.96 | **0.007^b^** |  |
|  | rs11886868  (Minor allele: C) | TT – 1  TC – 3  CC – 13 | Additive model | TC *vs* CC | TT – 10.8%  TC – 14.10±7.11  CC – 11.21±8.61 | 0.600^b^ |  |
|  |  |  | Dominant model | **NE** |  |  |  |
|  |  |  | Recessive model | CC *vs* TC + TT | CC – 11.21±8.61  TC + TT –13.27±6.04 | 0.665^b^ |  |
|  |  |  | Allelic model | C *vs* T | C – 11.76±8.21  T – 13.27±6.04 | 0.734^b^ |  |
|  |  |  | Overdominant model | TC *vs* CC + TT | TC – 14.10±7.11  CC + TT – 11.18±8.27 | 0.581^b^ |  |
| 2 (HU+) | rs4671393  (Minor allele: A) | GG – 14  GA – 10  AA – 2 | Additive model | AA *vs* AG *vs* GG | GG – 18.5 (3.7 – 32)^*^  GA – 17.65 (7.7 – 28.0)^*^  AA – 11.3 (6 – 16.6)^*^ | 0.512^c^ |  |
|  |  |  | Dominant model | GG *vs* GA + AA | GG – 18.5 (3.7-32)^*^  GA+AA – 16.45 (6.0 – 28.0)^*^ | 0.681^a^ |  |
|  |  |  | Recessive model | AA *vs* GG + GA | AA – 11.3 (6 – 16.6)^*^  GG + GA – 18.5 (3.7 – 32.0)^*^ | 0.248^a^ |  |
|  |  |  | Allelic model | A *vs* G | A – 16.63±6.65  G – 17.84±7.86 | 0.652^b^ |  |
|  |  |  | Overdominant model | GA *vs* GG + AA | GA – 17.7±6.34  GG+AA – 17.11±8.92 | 0.856^a^ |  |
|  | rs1427407  (Minor allele: T) | GG – 13  GT – 12  TT – 1 | Additive model | GT *vs* GG | GG – 18.5±8.48  GT – 17.1±7.06 | 0.295^b^ |  |
|  |  |  | Allelic model | T *vs* G | T – 16.21±7.42  G – 17.79±7.70 | 0.550 |  |
|  |  |  | Recessive model | **NE** |  |  |  |
|  |  |  | Dominant model | GG *vs* GT + TT | GG – 18.46±8.46  GT+TT – 16.21±7.42 | 0.481^b^ |  |
|  |  |  | Overdominant model | GT *vs* GG + TT | GT – 17.07±7.06  GG+TT – 17.56±8.8 | 0.876^b^ |  |
|  | rs7557939  (Minor allele: G) | AA – 7  AG – 16  GG – 3 | Additive model | AA *vs* AG *vs* GG | AA – 18.64±8.44  GA – 17.37±8.09  GG – 14.10±7.25 | 0.722^d^ |  |
|  |  |  | Dominant model | AA *vs* GA + GG | AA – 18.64±8.44  GA + GG – 16.85±7.86 | 0.618^b^ |  |
|  |  |  | Recessive model | GG *vs* GA + AA | GG – 14.10±7.25  GA + AA – 17.76±8.02 | 0.462^b^ |  |
|  |  |  | Allelic model | A *vs* G | A – 17.76±8.04  G – 16.85±7.86 | 0.716^b^ |  |
|  |  |  | Overdominant model | GA *vs* GG + AA | GA – 17.37±8.09  GG + AA – 17.28±8.0 | 0.978^b^ |  |
|  | rs11886868  (Minor allele: C) | TT – 2  TC – 7  CC – 17 | Additive model | TT *vs* TC *vs* CC | TT – 9.0 (6.0 – 12.0)^*^  TC – 16.60 (7.7 – 32.0)^*^  CC – 19.0 (3.7 – 29.0)^*^ | 0.305^c^ |  |
|  |  |  | Dominant model | TT *vs* TC*+*CC | TT – 9.0 (6.0 – 12.0)^*^  TC+CC – 18.50 (3.7 – 32.0) | 0.124^a^ |  |
|  |  |  | Recessive model | CC *vs* TT*+*TC | CC – 17.95±7.31  TT+TC – 16.18±9.24 | 0.597^b^ |  |
|  |  |  | Allelic model | T *vs* C | T – 16.18±9.24  C – 18.03±7.77 | 0.567^b^ |  |
|  |  |  | Overdominant model | CT *vs* TT*+*CC | CT – 18.23±9.43  TT+CC – 17.00±7.51 | 0.734^b^ |  |
| 3 (Female) | rs4671393  (Minor allele: A) | GG – 14  GA – 8  AA – 1 | Additive model | GA *vs* GG | GG – 17.85±7.32  GA – 16.7±7.45  AA – 6.0% | 0.728^b^ |  |
|  |  |  | Dominant model | GG *vs* GA+AA | GG – 17.85±7.32  GA+AA – 15.51±7.83 | 0.474^b^ |  |
|  |  |  | Recessive model | **NE** |  |  |  |
|  |  |  | Allelic model | A *vs* G | A – 15.51±7.83  G – 17.43±7.21 | 0.516^b^ |  |
|  |  |  | Overdominant model | GA *vs* GG+AA | GA – 16.7±7.45  GG+AA – 17.1±7.69 | 0.914^b^ |  |
|  | rs1427407  (Minor allele: T) | GG – 13  GT – 9  TT – 1 | Additive model | GT *vs* GG | GG – 17.61±7.56  GT – 17.18±7.12  TT – 6.0% | 0.894^b^ |  |
|  |  |  | Dominant model | GG *vs* GT+TT | GG – 17.61±7.56  GT+TT – 16.06±7.57 | 0.632^b^ |  |
|  |  |  | Recessive model | **NE** |  |  |  |
|  |  |  | Allelic model | T *vs* G | T – 16.06±7.59  G – 17.43±7.21 | 0.627^b^ |  |
|  |  |  | Overdominant model | GT *vs* GG+TT | GT – 17.18±7.12  GG+TT – 16.79±7.90 | 0.903^b^ |  |
|  | rs7557939  (Minor allele: G) | AA – 9  GA – 9  GG – 5 | Additive model | AA *vs* GA *vs* GG | AA – 19.68±7.62  GA – 14.98±6.51  GG – 15.52±8.68 | 0.380^d^ |  |
|  |  |  | Dominant model | AA *vs* GA + GG | AA – 19.68±7.62  GA + GG – 15.17±7.02 | 0.161^b^ |  |
|  |  |  | Recessive model | GG *vs* GA + AA | GG – 15.52±8.68  GA + AA – 17.33±7.29 | 0.642^b^ |  |
|  |  |  | Allelic model | A *vs* G | A – 17.33±7.29  G – 15.17±7.02 | 0.406^b^ |  |
|  |  |  | Overdominant model | GA *vs* GG + AA | GA – 14.98±6.51  GG + AA – 18.19±7.95 | 0.323^b^ |  |
|  | rs11886868  (Minor allele: C) | TT – 2  TC – 5  CC – 16 | Additive model | TT *vs* TC *vs* CC | TT – 9.0 (6.0 – 12.0)^*^  TC – 12.1 (11.0 – 32.0)^*^  CC – 17.15 (3.5 – 29.5)^*^ | 0.246^c^ |  |
|  |  |  | Dominant model | TT *vs* CC + TC | TT – 9.0 (6.0 – 12.0)^*^  CC + TC – 16.3 (3.5 – 32.0)^*^ | 0.102^a^ |  |
|  |  |  | Recessive model | CC *vs* TC + TT | CC – 17.71±6.91  TC + TT – 15.16±8.83 | 0.461^b^ |  |
|  |  |  | Allelic model | T *vs* C | T – 15.16±8.83  C – 17.69±7.28 | 0.456^b^ |  |
|  |  |  | Overdominant model | TC *vs* CC + TT | CT – 17.62±9.27  CC + TT – 16.74±7.16 | 0.822^b^ |  |
| 3 (Male) | rs4671393  (Minor allele: A) | GG – 8  GA – 11  AA – 1 | Additive model | GG *vs* GA | GG – 13.06±10.78  GA – 12.64±8.28  AA – 16.6% | 0.92^b^ |  |
|  |  |  | Dominant model | GG *vs* GA+AA | GG – 13.06±10.78  GA+AA – 12.97±7.97 | 0.98^b^ |  |
|  |  |  | Recessive model | **NE** |  |  |  |
|  |  |  | Allelic model | A *vs* G | A – 12.97±7.97  G – 12.81±9.12 | 0.963^b^ |  |
|  |  |  | Overdominant model | GA *vs* GG+AA | GA – 12.64±8.28  GG+AA – 13.45±10.15 | 0.844^b^ |  |
|  | rs1427407  (Minor allele: T) | GG – 8  GT – 12  TT – 0 | Additive model | GT *vs* GG | GT – 11.53±8.52  GG – 14.29±10.10 | 0.527^b^ |  |
|  |  |  | Allelic model | T *vs* G | T – 12.15±8.40  G – 13.0±8.92 | 0.790^a^ |  |
|  |  |  | Recessive model | **NE** |  |  |  |
|  |  |  | Dominant model | **NE** |  |  |  |
|  |  |  | Overdominant model | **NE** |  |  |  |
|  | rs7557939  (Minor allele: G) | AA – 1  GA – 16  GG – 3 | Additive model | GA *vs* GG | AA – 7.40%  GA – 12.95±9.63  GG – 15.17±6.18 | 0.709^b^ |  |
|  |  |  | Dominant model | **NE** |  |  |  |
|  |  |  | Recessive model | GG *vs* GA + AA | GG – 15.16±6.18  GA + AA – 12.62±9.42 | 0.661^b^ |  |
|  |  |  | Allelic model | A *vs* G | A – 12.22±9.58  G – 13.30±9.07 | 0.736^a^ |  |
|  |  |  | Overdominant model | GA *vs* GG + AA | GA – 12.95±9.63  GG + AA – 13.22±6.37 | 0.958^b^ |  |
|  | rs11886868  (Minor allele: C) | TT – 1  TC – 5  CC – 14 | Additive model | TT *vs* TC *vs* CC | TT – 10.8%  TC – 16.36±8.98  CC – 11.96±9.25 | 0.371^c^ |  |
|  |  |  | Dominant model | **NE** |  |  |  |
|  |  |  | Recessive model | CC *vs* TT*+*TC | CC – 11.96±9.26  TT+TC – 15.43±8.34 | 0.440^b^ |  |
|  |  |  | Allelic model | C *vs* T | C – 13.12±9.15  T – 15.43±8.34 | 0.588^b^ |  |
|  |  |  | Overdominant model | TC *vs* TT*+*CC | TC – 16.36±8.98  TT+CC – 11.89±8.93 |  |  |

SD: Standard deviation; SNP: Single nucleotide polymorphism; Min: minimum value; Max: maximum value; NE: not estimated due to insufficient number of individuals in the group; vs: versus; n: number of individuals per genotype; ^a^Mann-Whitney test; ^b^Independent t-test; ^c^Kruskal-Wallis test; ^d^one way ANOVA. p*: significance of the post hoc test. Value in bold: significant p-value.

The table shows the analyses referring to the *BCL11A* gene SNPs applying different genetic models: additive model, dominant model, recessive model, and allelic model. The rows in the first column are divided according to the analysis criteria: 1 - comparison between HbF levels; 2 - comparison between groups that use or do not use HU; and 3 - comparison between males and females.

**Table S3** – Analysis of *HBS1L-MYB* intergenic region SNPs for each of the criteria.

| Criteria | SNP | n | Dominance model | | Median (Min–Max)*  Mean±SD of %HbF | p value |
| --- | --- | --- | --- | --- | --- | --- |
| 1 | rs4895441  (Minor allele: G) | AA – 31  AG – 9  GG – 3 | Additive model | AA *vs* AG *vs* GG | AA – 23.13±9.58  AG – 15.42±7.58  GG – 14.24±8.24 | 0.210^d^ |
|  |  |  | Dominant model | AA *vs* AG + GG | AA – 14.24±8.24  AG + GG – 17.35±8.40 | 0.275^b^ |
|  |  |  | Recessive model | GG *vs* AG + AA | GG – 23.13±9.58  AG + AA – 14.50±8.01 | 0.082^b^ |
|  |  |  | Allelic model | A *vs* G | G – 17.35±8.40  A – 14.50±8.01 | 0.291^a^ |
|  |  |  | Overdominant model | AG *vs* AA + GG | AG – 15.42±7.58  AA + GG – 15.02±8.59 | 0.900^b^ |
|  | rs9402686  (Minor allele: A) | GG – 29  GA – 11  AA – 3 | Additive model | GG *vs* GA *vs* AA | GG – 14.18±8.52  GA – 15.35±6.80  AA – 23.13±9.58 | 0.208^d^ |
|  |  |  | Dominant model | GG *vs* GA+AA | GG – 14.18±8.52  GA +AA– 17.02±7.77 | 0.299^b^ |
|  |  |  | Recessive model | AA *vs* GA + GG | AA – 23.13±9.57  GA + GG – 14.50±8.01 | 0.082^b^ |
|  |  |  | Allelic model | G *vs* A | G – 14.50±8.01  A – 17.02±7.79 | 0.313^b^ |
|  |  |  | Overdominant model | GA *vs* AA + GG | GA – 15.35±6.80  AA + GG – 15.02±8.86 | 0.910^b^ |
|  | rs11759553  (Minor allele: T) | AA – 21  AT – 17  TT – 5 | Additive model | AA *vs* AT *vs* TT | AA – 12.25±8.04  AT – 17.43±7.96  TT – 19.22±7.77 | 0.077^d^ |
|  |  |  | Dominant model | AA *vs* AT+TT | AA – 12.25±8.04  AT+TT – 17.83±7.77 | **0.025^b^** |
|  |  |  | Recessive model | TT *vs* AT+AA | TT – 19.22±7.77  AT+AA – 14.57±8.31 | 0.243^b^ |
|  |  |  | Allelic model | A *vs* T | A – 14.56±8.31  T – 17.84±7.77 | 0.128^a^ |
|  |  |  | Overdominant model | AT *vs* AA + TT | AT – 17.30 (5.90 – 32.0) ^*^  AA + TT – 12.1 (2.0 – 29.50) | 0.143^a^ |

| 2 (HU-) | rs4895441  (Minor allele: A) | GG - 1  GA – 6  AA – 10 | Additive model | AA *vs* GA | AA – 10.40±8.62  AG – 13.80±7.73  GG – 12.1% | 0.442^b^ |
| --- | --- | --- | --- | --- | --- | --- |
|  |  |  | Recessive model | AA *vs* AG*+*GG | AA – 7.40 (2.0 – 29.5)^*^  AG+GG – 12.10 (5.90 – 26.60)^*^ | 0.344^a^ |
|  |  |  | Dominant model | **NE** |  |  |
|  |  |  | Allelic model | A *vs* G | G – 13.56±7.09  A – 11.67±8.21 | 0.605^b^ |
|  |  |  | Overdominant model | AG *vs* AA*+*GG | AG – 13.90±7.73  AA+GG – 10.55±8.19 | 0.439^b^ |
|  | rs9402686  (Minor allele: A) | GG – 9  GA – 7  AA – 1 | Additive model | GG *vs* GA | GG – 9.54±8.79  GA – 14.41±7.06  AA – 12.1% | 0.252^c^ |
|  |  |  | Dominant model | GG *vs* GA+AA | GG – 6.60 (2.0 – 29.5)^*^  GA+AA – 12.1 (5.9 – 24.6)^*^ | 0.136^a^ |
|  |  |  | Recessive model | **NE** |  |  |
|  |  |  | Allelic model | G *vs* A | A – 11.67±6.59  G – 14.12±8.21 | 0.472^b^ |
|  |  |  | Overdominant model | GA *vs* GG+AA | GA – 14.41±7.06  GG+AA – 9.80±8.33 | 0.251^b^ |
|  | rs11759553  (Minor allele: T) | AA – 8  AT – 8  TT – 1 | Additive model | AA *vs* AT | AA – 9.91±9.33  AT – 13.44±7.10  TT – 12.1% | 0.409^b^ |
|  |  |  | Dominant model | AA *vs* AT+TT | AA – 5.95 (2.0 – 29.5)^*^  AT+TT – 12.10 (5.9 – 24.6)^*^ | 0.229^b^ |
|  |  |  | Recessive model | **NE** |  |  |
|  |  |  | Allelic model | A *vs* T | A – 11.67±8.21  T – 13.29±6.65 | 0.620^b^ |
|  |  |  | Overdominant model | AT *vs* AA+TT | AT – 11.40 (5.9 – 24.6)^*^  AA+TT – 7.40 (2.0 – 29.5)^*^ | 0.290^a^ |
| 2 (HU+) | rs4895441  (Minor allele: G) | AA – 21  AG – 3  GG – 2 | Additive model | AA *vs* AG *vs* GG | AA – 16.30 (3.7 – 32.0)^*^  AG – 19.0 (11.0 – 26.0)^*^  GG – 28.65 (28.0 – 29.3)^*^ | 0.114^c^ |
|  |  |  | Allelic model | A *vs* G | A – 16.39±7.45  G – 22.66±7.63 | 0.100^b^ |
|  |  |  | Dominant model | AA *vs* AG*+*GG | AA – 16.07±7.59  AG+GG – 22.66±7.63 | 0.094^b^ |
|  |  |  | Recessive model | GG *vs* AA*+*AG | GG – 28.60±0.92  AA+AG – 16.45±7.46 | **0.032^b^** |
|  |  |  | Overdominant model | AG *vs* AA*+*GG | AG – 18.67±7.50  AA+GG – 17.16±8.09 | 0.763^b^ |
|  | rs9402686  (Minor allele: A) | GG – 20  GA – 4  AA – 2 | Additive model | GG *vs* GA *vs* AA | GG – 16.45 (3.7 – 32.0)^*^  GA – 15.50 (11.0 – 26.0)^*^  AA – 28.65 (28.0 – 29.30)^*^ | 0.127^c^ |
|  |  |  | Allelic model | G *vs* A | G – 16.39±7.46  A – 20.88±8.10 | 0.204^b^ |
|  |  |  | Recessive model | AA *vs* GG*+*GA | AA – 28.65 (28.0-29.3)^*^  GG+GA – 16.45 (3.7-32.0)^*^ | **0.04^a^** |
|  |  |  | Dominant model | GG *vs* GA*+*AA | GG – 16.27±7.72  GA+AA – 20.88±8.09 | 0.216^b^ |
|  |  |  | Overdominant model | GA *vs* GG*+*AA | GA – 17.0±6.98  GG+AA – 17.39±8.20 | 0.929^b^ |
|  | rs11759553  (Minor allele: T) | AA – 13  AT – 9  TT – 4 | Additive model | AA *vs* AT *vs* TT | AA – 13.68±7.14  AT – 20.98±7.25  TT – 21.0±7.70 | 0.055^b^ |
|  |  |  | Dominant model | AA *vs* AT+TT | AA – 13.68±7.14  AT+TT – 20.98±7.06 | **0.015^b^** |
|  |  |  | Recessive model | TT *vs* AT+AA | TT – 21.0±7.70  AT+AA – 16.67±7.92 | 0.322^b^ |
|  |  |  | Allelic model | A *vs* T | A – 16.67±7.92  T – 20.98±7.62 | 0.115^b^ |
|  |  |  | Overdominant model | AT *vs* AA + TT | AT – 20.98±7.25  AA + TT – 15.40±7.72 | 0.087^b^ |
| 3 (Female) | rs4895441  (Minor allele: G) | AA – 16  AG – 6  GG – 1 | Additive model | AA *vs* AG | AA – 16.42±8,0  AG – 19.12±6.33  GG – 12.1% | 0.468^b^ |
|  |  |  | Dominant model | AA *vs* AG+GG | AA – 16.42±8.0  AG+GG – 18.11±6.36 | 0.626^b^ |
|  |  |  | Recessive model | **NE** |  |  |
|  |  |  | Allelic model | A *vs* G | G – 18.11±6.36  A – 17.15±7.54 | 0.764^b^ |
|  |  |  | Overdominant model | AG *vs* AA+GG | AG – 19.12±6.34  AA+AG – 16.16±7.81 | 0.416^b^ |
|  | rs9402686  (Minor allele: A) | GG – 15  GA – 7  AA – 1 | Additive model | GG *vs* GA | GG – 16.71±8.19  GA – 18.10±6.38  AA – 12.1% | 0.698^b^ |
|  |  |  | Dominant model | GG *vs* GA*+*AA | GG – 16.71±8.19  GA+AA – 17.35±6.28 | 0.850^b^ |
|  |  |  | Recessive model | **NE** |  |  |
|  |  |  | Allelic model | G *vs* A | A – 17.35±6.28  G – 17.15±7.54 | 0.948^b^ |
|  |  |  | Overdominant model | GA *vs* GG*+*AA | GA – 18.10±6.38  GG+AA – 16.42±8.0 | 0.630^b^ |
|  | rs11759553  (Minor allele: T) | AA – 11  AT – 8  TT – 4 | Additive model | AA *vs* AT *vs* TT | AA – 14.30±7.12  AT – 20.51±7.42  TT – 17.02±6.95 | 0.204^d^ |
|  |  |  | Dominant model | AA *vs* AT+TT | AA – 14.30±7.12  AT+TT – 19.35±7.15 | 0.105^b^ |
|  |  |  | Recessive model | TT *vs* AT+AA | TT – 17.02±6.95  AT+AA – 16.91±7.72 | 0.979^b^ |
|  |  |  | Allelic model | A *vs* T | A – 16.91±7.72  T – 19.35±7.15 | 0.386^b^ |
|  |  |  | Overdominant model | AT *vs* AA + TT | AT – 20.51±7.42  AA + TT – 15.03±6.94 | 0.092^b^ |
| 3 (Male) | rs4895441  (Minor allele: G) | AA – 15  AG – 3  GG – 2 | Additive model | AA *vs* AG *vs* GG | AA – 8.20 (2.0 – 29.0)^*^  AG – 7.40 (5.9 – 10.80)^*^  GG – 28.65 (28.0 – 29.30)^*^ | 0.089^c^ |
|  |  |  | Dominant model | AA *vs* AG*+*GG | AA – 11.91±8.10  AG+GG – 16.28±11.44 | 0.357^b^ |
|  |  |  | Recessive model | GG *vs* AA*+*AG | GG – 28.65 (28.0 – 29.30)^*^  AA+AG – 7.95 (2.0 – 29.0) | **0.032**^a^ |
|  |  |  | Allelic model | A *vs* G | G – 16.28±11.44  A – 11.27±7.54 | 0.252^b^ |
|  |  |  | Overdominant model | GA *vs* AA*+*GG | GA – 8.03±2.51  AA+GG – 13.88±9.40 | 0.310^b^ |
|  | rs9402686  (Minor allele: A) | GG – 14  GA – 4  AA – 2 | Additive model | GG *vs* GA *vs* AA | GG – 7.55 (2.0 – 29.0)^*^  GA – 9.50 (5.9 – 17.3)^*^  AA – 28.65 (28 – 29.3)^*^ | 0.099^c^ |
|  |  |  | Dominant model | GG *vs* GA*+*AA | GG – 11.47±8.28  GA+AA – 16.58±10.10 | 0.251^b^ |
|  |  |  | Recessive model | AA *vs* GG*+*GA | AA – 28.65 (28.0 – 29.3)^*^  GG+GA – 7.95 (2.0 – 29.0)^*^ | **0.038^a^** |
|  |  |  | Allelic model | G *vs* A | A – 16.58±10.10  G – 11.27±7.55 | 0.183^a^ |
|  |  |  | Overdominant model | GA *vs* GG*+*AA | GA – 9.5 (5.9 – 17.3)  GG+AA – 10.6 (2.0 – 29.3) | 0.189^a^ |
|  | rs11759553  (Minor allele: T) | AA – 10  AT – 9  TT – 1 | Additive model | AA *vs* AT | AA – 9.99±8.73  AT – 14.69±7.78  TT – 28% | 0.234^b^ |
|  |  |  | Dominant model | AA *vs* AT+TT | AA – 9.99±8.73  AT+TT – 16.02±8.46 | 0.134^b^ |
|  |  |  | Recessive model | **NE** |  |  |
|  |  |  | Allelic model | A *vs* T | A – 12.22±8.42  T – 16.02±8.46 | 0.258^a^ |
|  |  |  | Overdominant model | AT *vs* AA+TT | AT – 14.69±7.78  AA+TT – 11.63±9.91 | 0.406^b^ |

SD: Standard deviation; SNP: Single nucleotide polymorphism; Min: minimum value; Max: maximum value; NE: not estimated due to insufficient number of individuals in the group; vs: versus; n: number of individuals per genotype; ^a^Mann-Whitney test; ^b^Independent t-test; ^c^Kruskal-Wallis test; ^d^one way ANOVA. Value in bold: significant p-value.

The table shows the analyses referring to the *HBS1L-MYB* intergenic region SPNs applying different genetic models: additive model, dominant model, recessive model, and allelic model. The rows in the first column are divided according to the analysis criteria: 1 - comparison between HbF levels; 2 - comparison between groups that use or do not use HU; and 3 - comparison between males and females.
